# Livestock Grazing Management Influences Survival of Giant and Red Pandas in Southwest China

**DOI:** 10.1002/ece3.72106

**Published:** 2025-09-18

**Authors:** Yanshan Zhou, Xiang Yu, Chao Chen, Wenlei Bi, Rui Ma, Jiabin Liu, Zushen Li, Guanwei Lan, Rong Hou, Haijun Gu, Xiaodong Gu, Jinke Zeng, Minghua Chen, Buqie Shang, Zuofu Xiang, Dunwu Qi

**Affiliations:** ^1^ College of Life and Environmental Sciences Central South University of Forestry & Technology Changsha Hunan China; ^2^ The Conservation of Endangered Wildlife Key Laboratory of Sichuan Province, Chengdu Research Base of Giant Panda Breeding Chengdu Sichuan China; ^3^ College of Forestry Central South University of Forestry & Technology Changsha Hunan China; ^4^ Sichuan Forestry and Grassland Bureau Chengdu Sichuan China; ^5^ Sichuan Meigu Dafengding National Nature Reserve Sichuan China

**Keywords:** activity range, giant pandas, habitat preference, habitat utilization, red pandas

## Abstract

Despite intensified global efforts in wildlife conservation, livestock grazing remained a critical factor driving habitat changes. The quantitative studies specifically addressing the impact of grazing on the habitat changes of giant ( *Ailuropoda melanoleuca* ) and red pandas (*Ailurus styani*) were notably scarce. To address this knowledge gap, we used grazing data, environmental variables, and animal locations to evaluate the habitat preferences and spatial utilization patterns of giant and red pandas. The research results indicated that the preferred habitat of giant pandas progressively contracted as grazing intensity increased, whereas that of red pandas gradually expanded. However, the habitat suitability index (HSI) for both species declined. Furthermore, the spatial overlap degree in preferred habitats and the contribution of grazing factors in the habitat evaluation model progressively increased as grazing intensity increased. Our research also revealed that livestock was predominantly distributed in the marginal areas with the giant and red pandas. To mitigate grazing disturbance, giant and red pandas exhibited divergent habitat selection patterns. Specifically, red pandas tended to favor lower‐altitude areas with steeper terrain under grazing pressure; giant pandas preferred steeper areas regardless of altitude. This study confirmed that grazing was a critical factor influencing the habitat selection of both giant and red pandas. Mitigating grazing pressure through targeted management interventions could significantly reduce this impact, offering a viable strategy for enhancing habitat conservation in the future. Our findings underscored the critical importance for wildlife protection departments in southwestern China to exercise greater caution when formulating and implementing grazing policies.

## Graphical abstract

Grazing activities significantly influence the habitat selection of both giant and red pandas. To mitigate grazing disturbance, giant pandas and red pandas exhibited divergent habitat selection patterns. Enhancing grazing management practices could effectively reduce the survival pressures faced by giant pandas and red pandas.

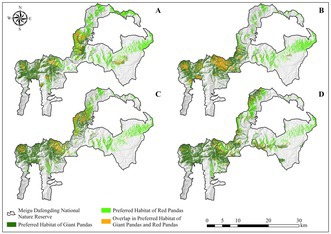

## 1 Introduction

At present, global biodiversity conservation is confronted with significant challenges (Llorente‐Culebras et al. 2023; Dong et al. 2024). Rapid economic development has profoundly impacted wild animals and their habitats (White et al. 2022), primarily through habitat loss and reduced distribution ranges (Ferreira et al. 2023), leading to a decline in the number of threatened wildlife species worldwide (Thakur et al. 2024). Since the 1960s, the Chinese government has established 67 nature reserves dedicated to giant pandas, significantly contributing to the prevention of further habitat degradation for both giant pandas and other co‐occurring wildlife species (Zhou et al. 2021; Ying et al. 2023; Dai et al. 2023). However, traditional practices of natural resource utilization, particularly livestock grazing, continue to occur in certain reserves, resulting in severe environmental degradation that threatens wildlife survival (Xiang et al. 2024). Therefore, understanding the factors influencing wildlife habitat selection, including both environmental and anthropogenic factors, is essential for ecological research (Martineau et al. 2016). Information on preferred habitat characteristics and potential threats is essential for developing effective wildlife management strategies (Shah et al. 2020).

The giant pandas ( *Ailuropoda melanoleuca* ) and red pandas (*Ailurus styani*) in western China are primarily distributed along the eastern edge of the Hengduan Mountains and in the adjacent forested mountainous regions (Ruan et al. 2024). Despite this, detailed information on their habitat selection preferences, spatial distribution patterns, and adaptation strategies under human disturbance remained limited (Zhang et al. 2022; Tang et al. 2022; Pema et al. 2020; Thapa et al. 2020). Due to concerted conservation efforts in China and globally over the past decades, the conservation status of the giant pandas in the IUCN Red List has been downgraded from “endangered” to “vulnerable”. In contrast, the red pandas (including two species) remained classified as “endangered” (Zang et al. 2017; Sikha et al. 2021). The conservation of both giant pandas and red pandas has been formally integrated into China's relevant legal frameworks (Wei et al. 2015; Ruan et al. 2024). The increasing fragmentation of habitats and the ongoing decline in population sizes of giant and red pandas have resulted in the formation of localized populations, which are now under significant survival pressure (Yang et al. 2018; Ruan et al. 2024). Current research indicated that traditional livestock grazing was a primary driver of habitat loss and population decline for giant and red pandas, particularly in the Liangshan Mountains of China (Zhang, Hull, et al. 2017; Li et al. 2019; Lama et al. 2020; Zhang et al. 2022). Grazing was a widespread disturbance activity that impacted multiple species (Bai et al. 2018; Hull et al. 2014; Kang et al. 2011; Wei et al. 2018). Livestock grazing has led to a rapid decline in shrub and bamboo populations, thereby degrading habitat quality (Ran et al. 2003; Ju et al. 2024). Additionally, increased grazing intensity has significantly altered the wildlife habitat preferences and activity ranges, imposing considerable negative impacts on their survival (Zhang, Li, et al. 2017).

Previous studies on wildlife habitat preferences have predominantly relied on broad‐scale disturbance factors, including human activity indices, economic development, mining, village presence, and road networks (Martineau et al. 2016; Zhang et al. 2022), with limited incorporation of detailed data on fine‐scale grazing practices (such as the distance from cattle stations, etc.) (Lama et al. 2020). Incorporating the distance from grazing sites as an independent variable in models assessing wildlife habitat preferences helps determine whether the foraging behavior of herbivores influences animal habitat use (Lama et al. 2020; Bista, Baxter, Hudson, Lama, and Murray 2021). However, including too many factors in habitat assessment models can lead to ambiguous results (Liu et al. 2024). Although some studies have examined the impacts of human activities on the habitats of giant and red pandas (Li et al. 2019; Bista et al. 2023), few studies have quantified the effects of grazing on their habitat preferences at comparable temporal and spatial scales. Historically, conservation efforts have disproportionately focused on giant pandas, leading to the relative neglect of red pandas in conservation initiatives. To address this knowledge gap, we propose the following research hypotheses: (1) Grazing situation will differentially affect the habitat preferences of giant and red pandas. (2) Given the greater vigilance of red pandas relative to giant pandas (Thapa et al. 2018), the two species are expected to exhibit distinct behavioral responses to the grazing situation (Bista et al. 2024). To test these hypotheses, we used detailed grazing data and targeted environmental factors to simultaneously evaluate the interactions between habitat preferences, spatial distribution, and grazing activities for both species. This study aims to provide a scientific foundation for the development of conservation strategies for wildlife species such as the giant and red pandas.

## 2 Materials and Methods

### 2.1 Study Area

**FIGURE 1:**
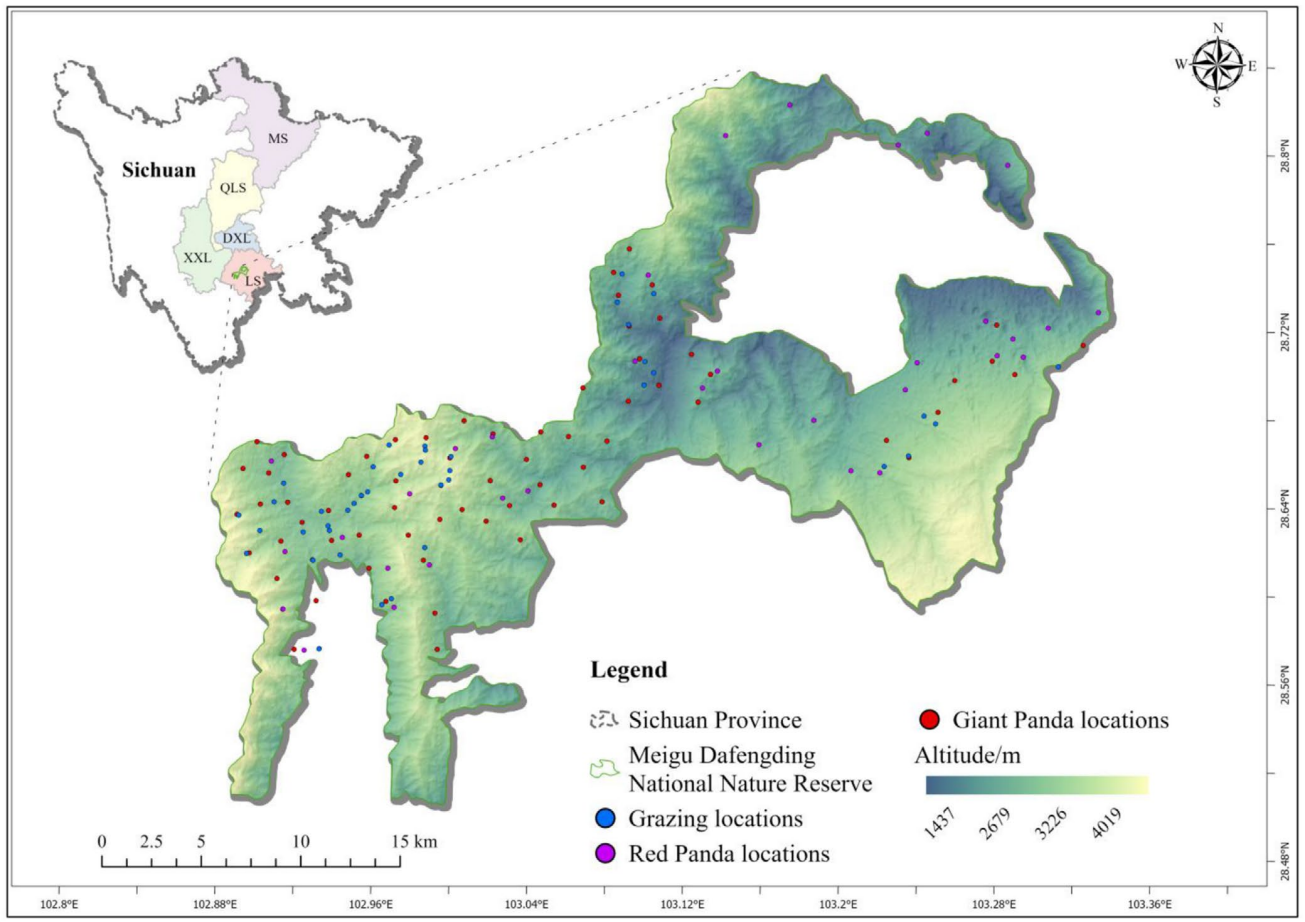
The study area was the Meigu Dafengding National Nature Reserve, located in southwestern China.

We selected the Meigu Dafengding National Nature Reserve in Sichuan Province, China (102°52′‐103°20′ E, 28°30′‐28°50′ N) as our study area (Figure 1). The reserve covers an area of 506.55 km^2^ and is one of the key distribution areas for both giant and red pandas within the Liangshan Mountain of China (Mao et al. 2023). This region is recognized as a global biodiversity hotspot, characterized by subtropical evergreen broad‐leaved forests, with elevations ranging from 1356 to 3998 m (Mao et al. 2023). The area is rich in bamboo species, which form an essential part of the diet for both giant and red pandas. The bamboo species include *Bashania fangiana*, *Yushania chungii*, *Chimonobambusa szechuanensis*, *Yushania lineolata*, *Qiongzhuea macrophylla*, *Q. tumidinoda*, *Indocalamus latifolius*, and *Chimonobambusa pachystachys* (Fu et al. 2020).

We chose the Meigu Dafengding National Nature Reserve for the following three reasons: Firstly, existing research indicates that this area is a key distribution zone for both giant and red pandas in China (State Forestry Administration 2015). Secondly, several counties within the Liangshan Mountain range remain classified as underdeveloped regions. In 2023, the per capita GDP in eight of these counties fell below 50% of the provincial average (Wang and Li 2024). Residents with lower economic incomes rely on natural resource use practices such as grazing, which inevitably influence the utilization of wildlife habitats (Li et al. 2019). Thirdly, due to environmental degradation, the local government has implemented a series of conservation policies, including restrictions on grazing and harvesting in certain areas. These factors suggest that human activities might significantly increase the extinction risk for these two species, thereby necessitating enhanced research and conservation efforts (State Forestry Administration 2015).

### 2.2 Field Investigation

We conducted surveys of giant and red pandas in the Meigu Dafengding National Nature Reserve during the spring (March to May) and autumn (October to November) of 2024. The field investigations were carried out using a 2 km^2^ grid system, collecting data on the locations of giant pandas, red pandas, and livestock within each grid (Table S1). The livestock in the study area consists of yaks ( *Bos grunniens* ), yellow cattle ( *Bos taurus* ), horses ( *Equus caballus* ), goats ( *Capra hircus* ) and sheep ( *Ovis aries* ). Giant and red pandas' locations were identified through feces, feeding signs, and footprints found within the survey grids, while grazing was identified by the presence of livestock, livestock feces, and trampling marks. No other forms of human disturbance were observed during this survey.

### 2.3 Evaluation of Giant and Red Pandas Preferred Habitat

We used the MaxEnt model to evaluate the habitat selection preferences of giant and red pandas. The model predicted the habitat suitability index (HSI) ranging from 0 (no preference) to 1 (high preference). The MaxEnt model has emerged as a widely recognized tool for predicting species' potential distributions (Fourcade et al. 2014; Kramer‐Schadt et al. 2013). Compared to other modeling approaches, MaxEnt employed the area under the receiver operating characteristic curve (AUC) for model evaluation and has been demonstrated to generate highly accurate predictions (Elith et al. 2006). In this study, we integrated environmental variables (including vegetation type, altitude, aspect, slope, and climatic factors) and disturbance variables (such as distance to the grazing locations and roads) into the MaxEnt model. This approach allowed us to assess the habitat preferences of giant and red pandas and evaluate the influence of these factors on their habitat selection (Phillips et al. 2006).

#### 2.3.1 Environment Variables and Grazing Activity

Forests were essential components of the habitats for both giant and red pandas (Zhang et al. 2006). However, giant pandas generally preferred relatively gentle slopes in mid‐altitude areas, whereas red pandas tended to favor steeper slopes in high‐altitude regions (Bista et al. 2019; Bai et al. 2020). Therefore, we acquired a comprehensive set of environmental variables for habitat prediction. This dataset included raster data on vegetation cover types, a digital elevation model (DEM) to describe altitude, and slope and aspect variables derived from the DEM (Viña et al. 2011). Vegetation types were determined through supervised classification using 30 m resolution Landsat TM remote sensing images, classifying coverage into grassland, shrubland, coniferous forest, broad‐leaved forest, and bare land (Dong et al. 2021). Given that forest presence, altitude, and slope have been shown to accurately predict habitats by giant and red pandas, we retained these variables in the original 30 m resolution raster data to prevent over‐smoothing of the prediction results (Dong et al. 2021). The climate data were obtained from the high‐resolution climate dataset (http://chelsaclimate.org/), which included 19 bioclimatic variables (BIO1‐BIO19) at a spatial resolution of approximately 1 km (Yang et al. 2017). To screen the 26 environmental variables (Table S2), we used the Pearson correlation coefficient and variance inflation factor (VIF). Variables with a correlation coefficient greater than 0.75 and VIF less than 0.5 were excluded (Dong et al. 2021). Ultimately, nine environmental variables were selected for inclusion in the model: altitude, aspect, slope, distance to grazing locations, distance to roads, distance to rivers, vegetation type, mean diurnal temperature range (Bio2), and annual precipitation (Bio12) (Table S3).

#### 2.3.2 Mapping Preferred Habitat of Giant and Red Pandas

To assess habitat preference selection, we analyzed the locations of 345 giant pandas and 44 red pandas identified in this study. To mitigate the potential impact of spatial autocorrelation on model prediction accuracy caused by closely spaced locations, we excluded red panda locations within 1.04 km and giant panda locations within 1.26 km based on their respective home range sizes (Dong et al. 2021). Consequently, 71 giant panda locations and 32 red panda locations were retained for model construction. We randomly selected 80% of the locations for model training and 20% for testing and repeated this process 10 times using the bootstrap method (Dong et al. 2021). We used the MaxEnt model to simulate habitat preference maps for giant and red pandas under different grazing situations by modeling environmental predictor variables across the entire study area. Specifically, we considered four situations: current grazing (including all grazing locations), moderate grazing (including 50% of random grazing locations), low grazing (including 25% of random grazing locations), and no grazing (excluding grazing locations). The selection of 50% and 25% random grazing locations was accomplished by the Create Random Points tool of GIS software. We categorized changes in habitat preference into two classes: preferred and non‐preferred (Yang et al. 2017). In the MaxEnt model incorporating both grazing and environmental variables, we assessed the relative importance of these factors by evaluating their contribution rates to the model, thereby estimating their influence on the habitat preferences of giant and red pandas. We evaluated the model's accuracy using the AUC value, considering it acceptable if the AUC exceeded 0.8 (Yang et al. 2017; Li et al. 2019). Based on the average HSI derived from 10 repetitions, we assessed the habitat preferences of giant and red pandas in the study area. Using the Maximum Training Sensitivity Plus Specificity logistic threshold, we reclassified the model results into preferred and non‐preferred habitats. Previous studies have demonstrated that grazing activities could influence the habitats of giant and red pandas (Lama et al. 2020; Zhang et al. 2022). To investigate the impact of grazing on the habitat quality for giant and red pandas, we extracted the HIS values of each grid center point and compared the HIS under current grazing, moderate grazing, low grazing, and no grazing conditions using the Kruskal‐Wallis test (the confidence interval is 95%). This approach allowed us to quantify the differences in habitat suitability between the two species.

### 2.4 Spatial Utilization of Habitat Preferences for Giant and Red Pandas

We analyzed changes in the preferred habitats of giant and red pandas at varying levels of spatial utilization. To differentiate between these levels, we first used location data for both species and applied Kernel Density Estimation (KDE) to estimate their distributions within the study area. KDE was a widely used method for delineating animal home ranges and could be applied at both individual and population levels (Hull et al. 2011). It relied on field‐collected data and employed non‐parametric estimation techniques to model spatial distribution, thereby delineating habitat ranges and quantifying spatial utilization density (Powell 2000). It was very important to select an appropriate smoothing coefficient (*h*) in KDE, as it would affect the final distribution output (Zhang et al. 2013). We used the LSCV (Least Squares Cross Validation) method to calculate the *h* value through likelihood cross‐validation (CVh), because previous studies have found that the *h* value calculated using CVh was closer to the actual utilization distribution and could provide more stable estimates when the sample size was small (Zhang et al. 2013). CVh calculation yielded *h* values of 0.063, 0.125, and 0.094 for giant pandas, red pandas, and livestock, respectively. We generated 95%, 50%, and 25% probability density contour lines for giant pandas, red pandas, and livestock locations. The 25% contour line was designated as the core distribution area, the region between 25% and 50% as the potential distribution area, and the region between 50% and 95% as the marginal distribution area (Li et al. 2019). Subsequently, we quantified the overlap between the core, potential, and marginal grazing distribution areas and the respective habitats of giant and red pandas. We then evaluated the impact of grazing events on these two species under current, moderate, and low grazing situations. All spatial analyses were conducted using ArcGIS 10.8.

### 2.5 Characteristics of Habitat Selection of Giant and Red Pandas Under Grazing Situations

We extracted habitat factors (altitude, slope, and aspect) for the locations of giant pandas, red pandas, and livestock from the DEM (Table S4). We used the Kruskal‐Wallis test (95% confidence level) to analyze the correlations between the habitat selection patterns of giant pandas, red pandas, and livestock, thereby elucidating the habitat selection characteristics of these two species in response to grazing. All statistical analyses were conducted using R software (version 4.3.1).

## 3 Results

### 3.1 Habitat Preferences of Giant and Red Pandas Under the Different Grazing Conditions

Under the different grazing conditions, the AUC value of the habitat assessment model ranged from 0.817 to 0.879 and 0.858 to 0.901 for the giant and red pandas, respectively (Table 1). These results suggested that our model demonstrated a high level of accuracy in predicting the preferred habitats of this species.

**TABLE 1:** The AUC values, preferred habitat area, and HIS for giant and red pandas under the different grazing conditions in Meigu Dafengding National Nature Reserve, China.

Species	Grazing condition	AUC value	Preferred habitat area (km^2^)	HSI			
				Value	df	Chi‐Square	*p*
Giant pandas	Current grazing	0.876	90.28	0.452 ± 0.083^A^	3	71582.20	< 0.0001
	Moderate grazing	0.863	102.46	0.466 ± 0.094^B^			
	Low grazing	0.871	102.62	0.475 ± 0.078^C^			
	No‐grazing	0.817	112.81	0.525 ± 0.077^D^			
Red pandas	Current grazing	0.870	99.57	0.490 ± 0.078^a^	3	41493.15	< 0.0001
	Moderate grazing	0.893	84.22	0.511 ± 0.072^c^			
	Low grazing	0.901	76.64	0.501 ± 0.080^b^			
	No‐grazing	0.858	69.76	0.541 ± 0.053^d^			

*Note:* Capital letters indicate significant differences in HSI of giant pandas under different grazing conditions, and lowercase letters indicate significant differences in HSI of red pandas under different grazing conditions.

The Maxent model predictions revealed significant variations in the preferred habitat area and Habitat Suitability Index (HSI) of giant and red pandas across different grazing conditions (Table 1). Specifically, the preferred habitat area of giant pandas increased as grazing intensity decreased (current grazing < moderate grazing < low grazing < no grazing). In contrast, the preferred habitat area of red pandas decreased with declining grazing intensity (current grazing > moderate grazing > low grazing > no grazing). Regarding HSI, both giant and red pandas exhibited a progressive increase as grazing intensity decreased (current grazing < moderate grazing < low grazing < no grazing). Taken together, although reduced grazing intensity led to an expansion of preferred habitat area for giant pandas and a contraction for red pandas (possibly because red pandas shifted to marginal zones under grazing pressure), the increasing trend in HSI for both species suggested that lowering grazing intensity contributed to improved habitat quality for both giant and red pandas.

**FIGURE 2:**
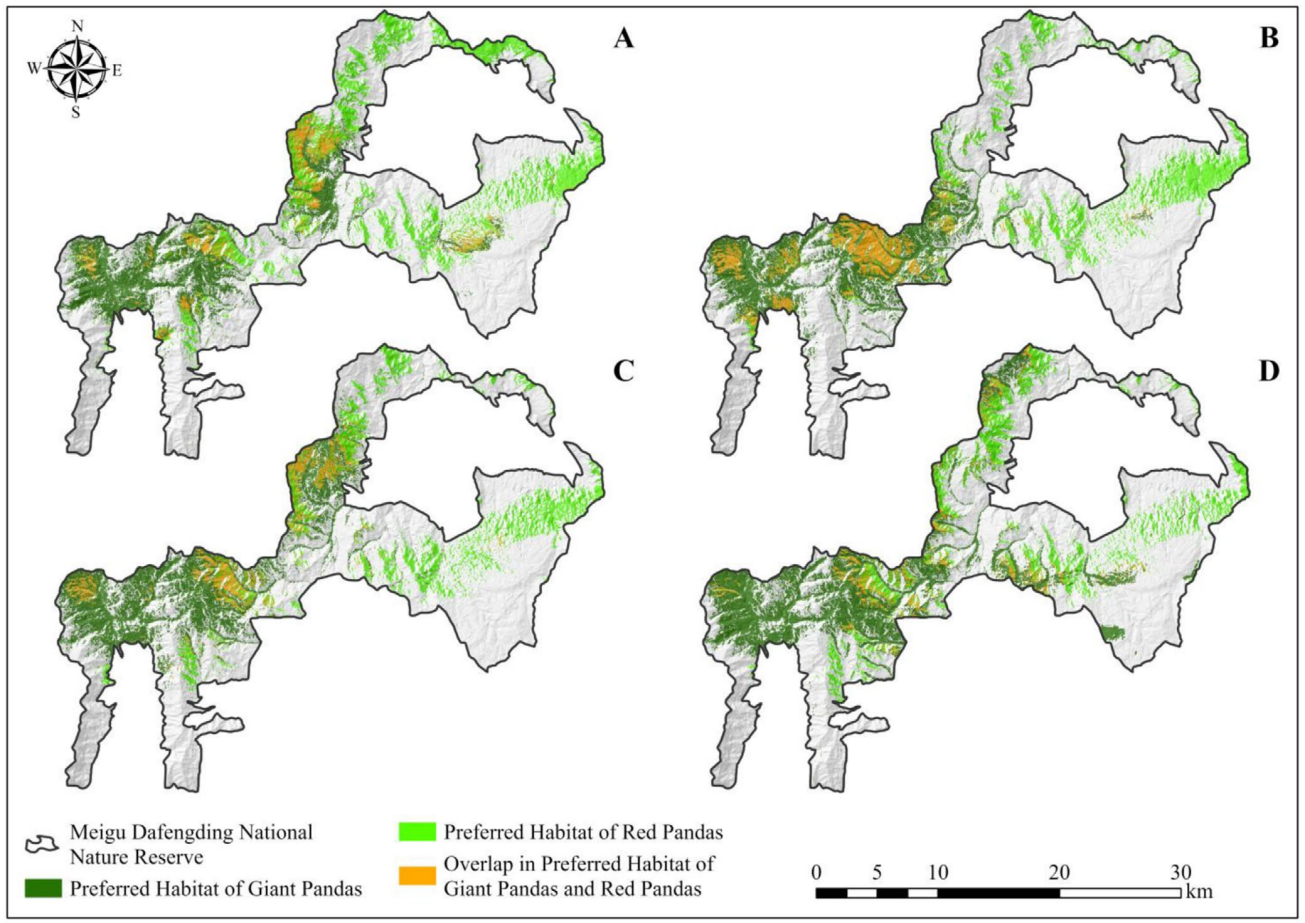
The preferred habitats of giant and red pandas under different grazing conditions in Meigu Dafengding National Nature Reserve, China. (A–D) showed the distribution of preferred habitats for both species under the current, moderate, low, and no‐grazing conditions, respectively.

Furthermore, our analysis revealed that under the current grazing condition, the overlapping area of preferred habitats for giant and red pandas is 25.03 km^2^ (Figure 2A). It is predicted that when grazing intensity decreases from the current level to moderate, the overlapping area of their preferred habitats would reduce to 17.88 km^2^ (Figure 2B). When grazing intensity further decreases to low levels, the overlapping area is expected to be 18.02 km^2^ (Figure 2C). In the most idealized non‐grazing condition, this overlapping area is projected to decrease to 13.26 km^2^ (Figure 2D). These findings suggest that the degree of overlap in preferred habitats between giant and red pandas tends to decrease as grazing intensity declines.

### 3.2 The Impact of Grazing on the Habitat Preferences of Giant and Red Pandas

**FIGURE 3:**
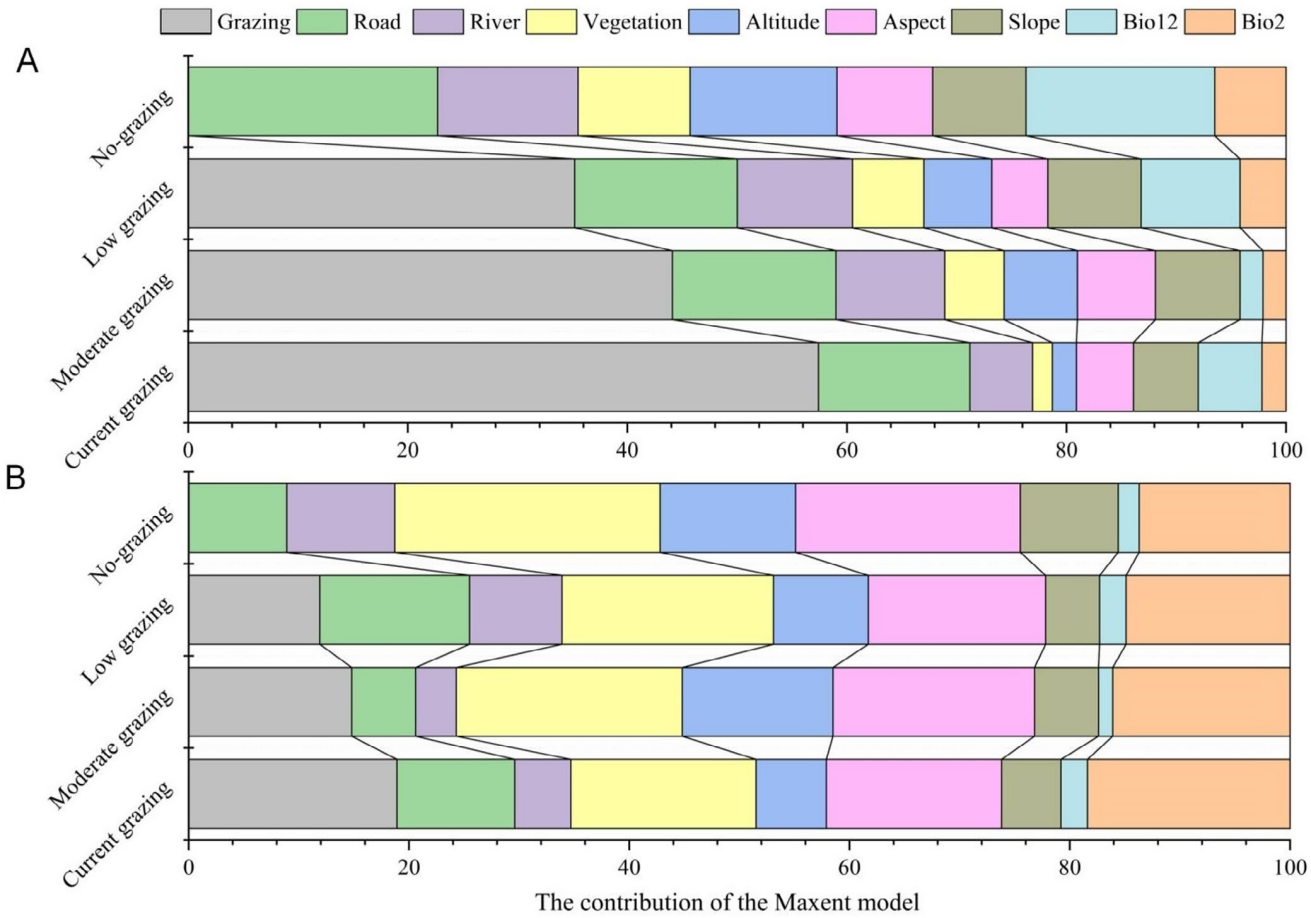
The importance of environmental variables in the Maxent model for giant and red pandas under different grazing conditions in Meigu Dafengding National Nature Reserve in Sichuan Province, China. A and B showed the contribution of environmental variables to the Maxent model for giant and red pandas under the current, moderate, low, and no‐grazing conditions, respectively.

Using the Maxent model to evaluate the preferred habitats of giant and red pandas, we found that grazing significantly influenced preferred habitat selection for both species. Specifically, under current, moderate, and low grazing intensities, the contribution rates of the grazing variable to the Maxent model for giant pandas were 57.3%, 44.1%, and 35.2%, respectively, showing a decreasing trend as grazing intensity decreased (Figure 3A, Table S5). Similarly, for red pandas, the contribution rates of the grazing variable were 18.9%, 14.8%, and 11.9% under the same grazing intensities, also showing a decreasing trend as grazing intensity decreased (Figure 3B, Table S5).

### 3.3 Spatial Utilization of Giant and Red Pandas Under Current Grazing Conditions

Based on the KDE analysis, the core, potential, and marginal distribution areas for giant pandas were 19.02 km^2^, 34.22 km^2^, and 140.36 km^2^, respectively, while those for red pandas were 25.08 km^2^, 70.51 km^2^, and 359.90 km^2^, respectively. These findings aligned with the differences in their preferred habitats.

**FIGURE 4:**
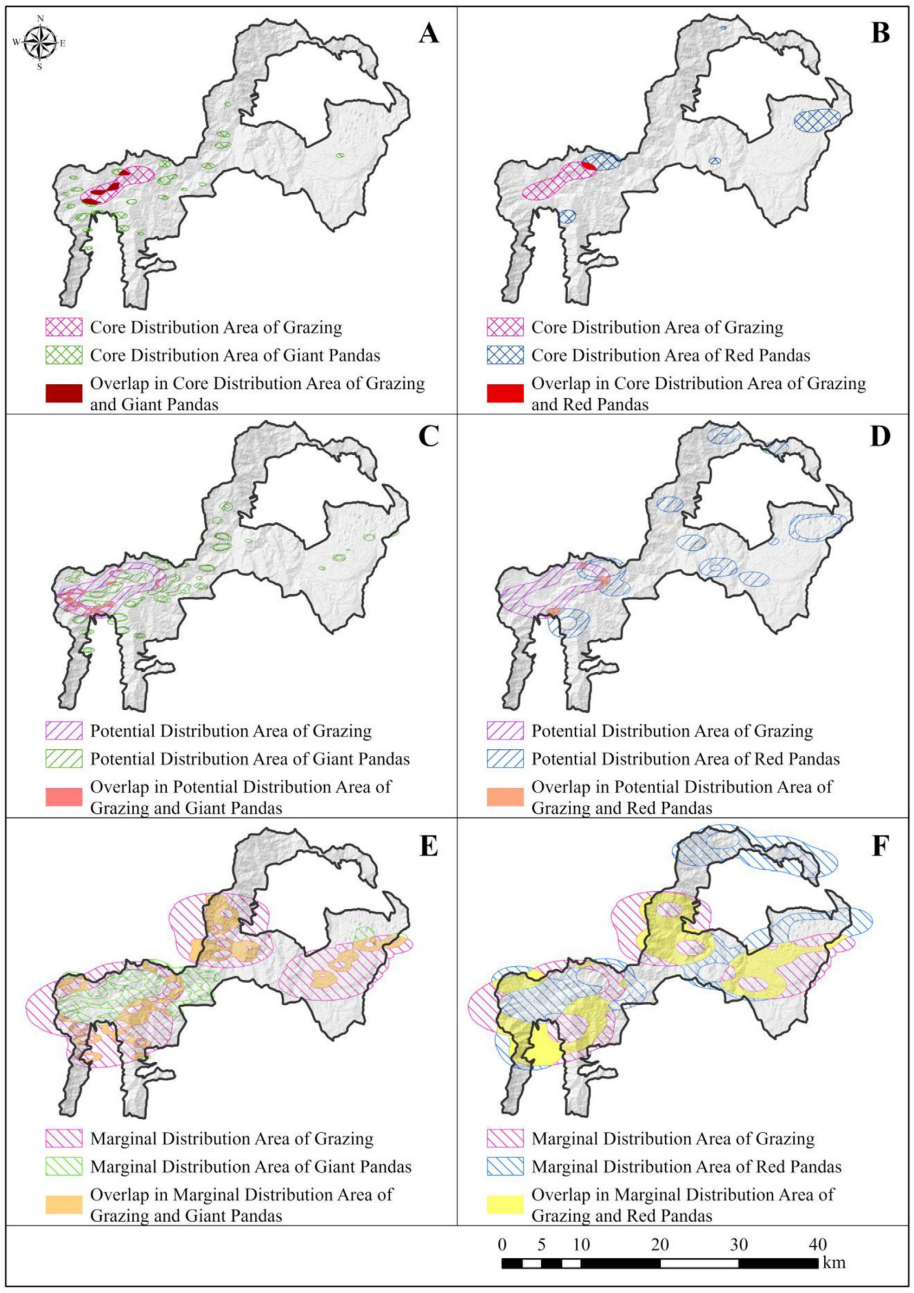
The spatial overlap between giant and red pandas with livestock in core, potential, and marginal distribution areas in Meigu Dafengding National Nature Reserve in Sichuan Province, China. (A, B) showed the spatial overlap of giant and red pandas with livestock in the core distribution area, respectively. (C) and (D) showed the spatial overlap of giant and red pandas with livestock in the potential distribution area, respectively. (E, F) showed the spatial overlap of giant and red pandas with livestock in the marginal distribution area, respectively.

Our research revealed that the overlapping area between the core distribution area of giant pandas and livestock was 3.79 km^2^, accounting for 19.93% of the giant pandas' core distribution area (Figure 4A). In contrast, the overlap for red pandas was only 1.16 km^2^, accounting for 4.61% of their core distribution area (Figure 4B). For potential grazing areas, the overlap with giant pandas was 7.04 km^2^, or 20.57% of their potential distribution area (Figure 4C), while for red pandas, it was 3.57 km^2^, representing 5.07% of their potential distribution area (Figure 4D). Similarly, in marginal grazing areas, the overlap with giant pandas was 85.42 km^2^, or 60.86% of their marginal distribution area (Figure 4E), whereas for red pandas, it was 169.12 km^2^, which accounts for 46.99% of their marginal distribution area (Figure 4F). These findings suggested that grazing activities predominantly occurred in the marginal distribution areas of both species, indicating a potential differentiation in habitat selection between giant pandas, red pandas, and livestock.

### 3.4 Habitat Selection Characteristics of Giant and Red Pandas Under Grazing Disturbance

**FIGURE 5:**
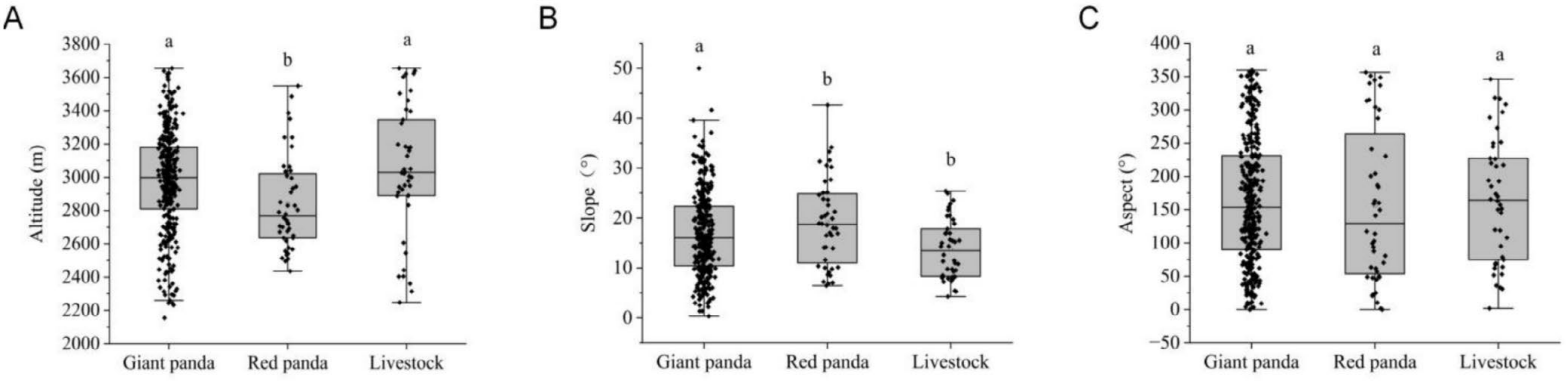
Analysis of differences in habitat selection among giant pandas, red pandas, and livestock under different grazing conditions in Meigu Dafengding National Nature Reserve in Sichuan Province, China. The error bars in the figure represent the standard deviation. Bars sharing different lowercase letters indicated statistically significant differences at *p* < 0.05. (A) showed that there was no significant difference in altitude utilization between giant pandas and livestock, while red pandas exhibited a significant preference for lower altitudes compared to livestock. (B) showed that both giant and red pandas demonstrate a significantly higher preference for steeper slopes than livestock. (C) showed that there was no significant difference in aspect utilization among giant pandas, red pandas, and livestock.

To further validate the above results, we conducted a statistical analysis of habitat characteristic differences between giant pandas, red pandas, and livestock. The analyses revealed no significant difference in altitude selection between giant pandas (2981.70 ± 304.09 m) and livestock (3045 ± 386 m) (df = 2, *H* = 13.05, *p* = 0.73). In contrast, red pandas (2840 ± 279 m) exhibited a preference for lower‐altitude areas (*f* = 2, *H* = 13.05, *p* = 0.003) (Figure 5A). Additionally, both giant pandas (17° ± 8°) and red pandas (19° ± 9°) showed significantly higher preferences for steeper slopes compared to livestock (14° ± 6°) (df = 2, *H* = 9.10, *p* < 0.05) (Figure 5B). However, there was no significant difference in aspect selection among giant pandas (164° ± 96°), red pandas (156° ± 115°), and livestock (165° ± 92°) (df = 2, *H* = 0.70, *p* > 0.05) (Figure 5C). These findings suggested that under current grazing disturbance, habitat selection has diverged between giant pandas, red pandas, and domestic livestock. Specifically, giant pandas exhibited differentiation primarily in slope preference, while red pandas showed differentiation in both altitude and slope.

## 4 Discussion

Most previous studies on habitats primarily focused on the abiotic factors of giant and red pandas, such as forest coverage, slope, and altitude, while important human activities like grazing were not fully integrated into habitat suitability models (Ning et al. 2014; Wei et al. 2018; Yang et al. 2017; Ruan et al. 2024). Notably, most studies have concentrated on flagship species like the giant panda, while other co‐distributed species, such as red pandas, have been overlooked (Dong et al. 2021). Given current external disturbances, it remained uncertain whether the “umbrella effect” of giant panda conservation had a positive impact on other species. Therefore, we investigated changes in habitat preferences and distribution of giant and red pandas under current grazing disturbances. Our results confirmed that grazing affected habitat quality differently: when grazing intensity decreased, the habitat area for giant pandas increased, whereas it decreased for red pandas. This finding added some nuances to the previous understanding. Existing studies have assumed that the effects of grazing on giant pandas and their sympatric species were the same (Bragina et al. 2015). In our study area, grazing events were more frequent than any other form of human interference. Thus, our research provided a solid foundation for quantifying grazing's impact on giant and red pandas outside the Meigu Dafengding Nature Reserve. To our knowledge, this was the first study to integrate grazing and environmental variables into habitat models to assess changes in habitat preferences at the same spatial scale, thereby quantifying the impact of grazing on these two species. Studying the different responses of co‐distributed species to grazing was crucial. Our findings highlight the risks of using a single “umbrella species” to represent diverse human impacts on wildlife (Tang et al. 2022), as different species respond variably to grazing methods, potentially leading to incorrect conclusions (Zhang, Hull, et al. 2017).

Under the current grazing conditions, our field surveys revealed that a significantly lower detection probability of red panda tracks compared to those of giant pandas (345 locations for giant pandas versus 44 for red pandas). These differences might be attributed to the distinct biological characteristics and resource requirements of the two species. Both giant and red pandas rely heavily on bamboo as their primary food source (Zhang et al. 2006). When grazing disturbances occur within or near dense bamboo forests, these animals may adjust their activity patterns or relocate to other areas to coexist with livestock (Carter et al. 2012; Bista, Baxter, Hudson, Lama, and Murray 2021; Bista, Baxter, Hudson, Lama, Weerman, and Murray 2021; Bista et al. 2023, 2024). Our study confirmed that giant and red pandas employed distinct habitat selection strategies in response to grazing events. Specifically, red pandas tended to inhabit areas at lower elevations with steeper slopes, whereas giant pandas showed a preference for moving to steeper terrain. Previous research indicated that giant pandas were most active during the morning, evening, and midnight (Zhang et al. 2015). In response to grazing disturbances, giant pandas might modify their daily activity patterns, concentrating feeding within shorter time frames and smaller areas. In contrast, red pandas found it more challenging to avoid grazing disturbances through simple adjustments to their daily routines and instead tended to relocate to other regions to avoid livestock interference. This finding aligned with our analysis, which showed that the preferred habitat area for giant pandas decreases as grazing intensity increases, while it increases for red pandas. Red pandas primarily consume tender bamboo shoots and leaves (Zhang et al. 2011; Zhang, Li, et al. 2017), which necessitates broader foraging ranges compared to those of giant pandas (Zhang, Hull, et al. 2017).

Human activities significantly impact the suitability and landscape connectivity of habitats for giant and red pandas (Bista, Baxter, Hudson, Lama, and Murray 2021; Bista, Baxter, Hudson, Lama, Weerman, and Murray 2021; Bista et al. 2023, 2024). Our research indicated that grazing played a crucial role in shaping the preferred habitats of these species, suggesting that grazing intensity was a significant factor influencing their habitat selection. As grazing intensity increased, the preferred habitat area for giant pandas decreased, whereas that for red pandas increased. However, our results also showed that grazing negatively affected habitat quality, as indicated by the declining HSI values for both species with increasing grazing intensity. Previous studies have demonstrated that red pandas preferred steeper areas (Hull et al. 2016), whereas giant pandas favored gentle or moderate slopes (Bai et al. 2020), which contributed to their coexistence (Bai et al. 2020; Thapa et al. 2020). Notably, red pandas exhibit higher alertness compared to other wildlife and respond more strongly to disturbances such as grazing, roads, and settlements in their habitat selection processes (Pema et al. 2020; Bista et al. 2023). After livestock intrusion, the activity areas of red pandas became less suitable, prompting them to expand outward. The degree of spatial overlap between red pandas and livestock distribution areas was smaller than that between giant pandas and livestock under current grazing conditions. This was partly because red pandas moved to areas with reduced forest cover and steeper slopes, which might indicate forced displacement into less suitable habitats. Additionally, both giant and red pandas tend to avoid areas with human activity when selecting habitats (Bai et al. 2020; Bista et al. 2019, 2023, 2024; Thapa et al. 2020; Bista, Baxter, Hudson, Lama, and Murray 2021; Bista, Baxter, Hudson, Lama, Weerman, and Murray 2021; Limbu et al. 2024). The livestock are primarily concentrated in the marginal distribution areas of both species under current grazing situations. The habitat model predictions from this study showed that as grazing intensity increased, the spatial overlap of preferred habitats between giant and red pandas gradually decreased. Whether protected species such as giant pandas can coexist with livestock in the long term requires further investigation. For instance, our findings revealed that under current grazing disturbance, the distribution range of red pandas was larger than that of giant pandas, with red pandas primarily expanding into areas outside grazing zones due to their lower overlap with livestock activity ranges. Through further in‐depth analysis of grazing disturbance factors, we identified several potential conservation concerns, including the possibility that giant and red pandas were actively avoiding livestock disturbances. In our study area, grazing was concentrated in the marginal distribution areas of giant and red pandas, while their core distributions remained distant from grazing events. In the long term, grazing represents a significant threat to the habitat quality of giant pandas and their sympatric species. Controlling human‐induced disturbances, particularly high‐impact activities such as grazing, is essential for the conservation of these species' habitats.

In most protected areas in western China, grazing activities were still increasing significantly (Li et al. 2019; Mao et al. 2023). Local residents mainly relied on livestock for their livelihoods. After the implementation of the policy of returning farmland to forest, they increasingly used livestock as their main source of income (Hull et al. 2011). A large number of studies have shown that giant and red pandas competed with livestock for food resources and habitats (Wang et al. 2015; Zhang, Hull, et al. 2017; Lama et al. 2020; Dong et al. 2021). Therefore, measures must be taken to strengthen grazing control. This study focused on examining the relationship between grazing intensity and species' habitat and distribution patterns; however, it did not conduct a systematic analysis of the mechanisms through which grazing intensity influences population dynamics. Future research should involve continuous monitoring of population changes in giant and red pandas within the existing grazing management framework. It is essential to strictly maintain livestock carrying capacity below the ecological threshold of the pasture, thereby preventing the expansion of livestock activity areas from negatively impacting wildlife populations. The Chinese government is implementing the construction of the Giant Panda National Park to integrate management resources for the conservation of the giant panda's habitat, which helps alleviate the local extinction threats caused by habitat fragmentation and human activities. However, the Liangshan Mountains have not been included in the scope of this park; grazing disturbance continues to represent a primary threat to wildlife in this region (Li et al. 2019). The inclusion of the Liangshan Mountains within the park's jurisdiction, coupled with the development of scientifically sound and practical grazing control measures, could significantly contribute to mitigating this threat. Our research emphasizes the substantial influence of grazing management on wildlife habitat selection and patterns of spatial utilization. This is particularly important for species with strict dietary requirements or high vigilance, as it may reduce their ability to coexist with humans on a smaller scale. These findings highlight the importance of separating human activities from conservation efforts in protected landscapes to achieve greater coexistence (Western et al. 2009; Bista, Baxter, Hudson, Lama, Weerman, and Murray 2021), such as moving grazing activities away from threatened wildlife habitats. Moreover, our research also emphasizes that when formulating policies aimed at jointly managing conservation and human well‐being, potential impacts on giant pandas and their sympatric species must be carefully considered, especially in protected areas where traditional natural resource utilization is crucial for human livelihoods.

## Author Contributions

**Yanshan Zhou:** conceptualization (equal), data curation (equal), formal analysis (equal), investigation (equal), methodology (equal), validation (equal), visualization (equal), writing – original draft (lead), writing – review and editing (lead). **Xiang Yu:** conceptualization (equal), data curation (equal), formal analysis (equal), validation (equal). **Chao Chen:** conceptualization (equal), data curation (equal), formal analysis (equal), investigation (equal). **Wenlei Bi:** conceptualization (equal), investigation (equal), methodology (equal), writing – review and editing (equal). **Rui Ma:** conceptualization (equal), formal analysis (equal), methodology (equal), supervision (equal). **Jiabin Liu:** investigation (equal), methodology (equal), project administration (equal), supervision (equal). **Zushen Li:** investigation (equal), methodology (equal), project administration (equal). **Guanwei Lan:** investigation (equal), methodology (equal), project administration (equal), validation (equal). **Rong Hou:** conceptualization (equal), funding acquisition (equal), methodology (equal), resources (equal), validation (equal). **Haijun Gu:** conceptualization (equal), data curation (equal), investigation (equal), supervision (equal). **Xiaodong Gu:** conceptualization (equal), data curation (equal), investigation (equal), supervision (equal). **Jinke Zeng:** data curation (equal), investigation (equal), supervision (equal), validation (equal). **Minghua Chen:** data curation (equal), investigation (equal), supervision (equal), validation (equal). **Buqie Shang:** data curation (equal), investigation (equal), supervision (equal), validation (equal). **Zuofu Xiang:** conceptualization (equal), supervision (equal), validation (equal), writing – original draft (supporting), writing – review and editing (supporting). **Dunwu Qi:** conceptualization (equal), funding acquisition (equal), supervision (equal), writing – original draft (supporting), writing – review and editing (supporting).

## Conflicts of Interest

The authors declare no conflicts of interest.

## Supporting information


**Data S1:** ece372106‐sup‐0001‐TableS1‐S5.docx.

## Data Availability

All the required data are uploaded as Supporting Information.

## References

[ece372106-bib-0001] Bai, W. , T. Connor , J. Zhang , et al. 2018. “Long‐Term Distribution and Habitat Changes of Protected Wildlife: Giant Pandas in Wolong Nature Reserve, China.” Environmental Science and Pollution Research 25: 11400–11408. 10.1007/s11356-018-1407-6. 29423692

[ece372106-bib-0002] Bai, W. , Q. Huang , J. Zhang , J. Stabach , and V. Hull . 2020. “Microhabitat Selection by Giant Pandas.” Biological Conservation 247: 108615. 10.1016/j.biocon.2020.108615.

[ece372106-bib-0003] Bista, D. , G. Baxter , N. Hudson , S. Lama , and P. Murray . 2021. “Effect of Disturbances and Habitat Fragmentation on an Arboreal Habitat Specialist Mammal Using GPS Telemetry: A Case of the Red Panda.” Landscape Ecology 37, no. 3: 795–809. 10.1007/s10980-021-01357-w. 34720409 PMC8542365

[ece372106-bib-0004] Bista, D. , G. Baxter , N. Hudson , S. Lama , J. Weerman , and P. Murray . 2021. “Movement and Dispersal of a Habitat Specialist in Human‐Dominated Landscapes: A Case Study of the Red Panda.” Movement Ecology 9: 62. 10.1186/s40462-021-00297-z. 34906253 PMC8670026

[ece372106-bib-0005] Bista, D. , G. Baxter , N. Hudson , S. Lama , J. Weerman , and P. Murray . 2024. “Red Pandas on the Move: Weather and Disturbance Effects on Habitat Specialists.” Wildlife Biology 2024: e01384. 10.1002/wlb3.01384.

[ece372106-bib-0006] Bista, D. , G. Baxter , N. Hudson , and P. Murray . 2023. “Seasonal Resource Selection of an Arboreal Habitat Specialist in a Human‐Dominated Landscape: A Case Study Using Red Panda.” Current Zoology 69: 1–11. 10.1093/cz/zoac014. 36974152 PMC10039176

[ece372106-bib-0007] Bista, D. , P. K. Paudel , S. R. Jnawali , A. P. Sherpa , S. Shrestha , and K. P. Acharya . 2019. “Red Panda Fine﹕Cale Habitat Selection Along a Central Himalayan Longitudinal Gradient.” Ecology and Evolution 9: 5260–5269. 10.1002/ece3.5116. 31110677 PMC6509368

[ece372106-bib-0008] Bragina, E. , V. Radeloff , M. Baumann , K. Wendland , T. Kuemmerle , and A. Pidgeon . 2015. “Effectiveness of Protected Areas in the Western Caucasus Before and After the Transition to Post‐Socialism.” Biological Conservation 184: 456–464. 10.1016/j.biocon.2015.02.013.

[ece372106-bib-0009] Carter, N. H. , B. K. Shrestha , J. B. Karki , N. M. B. Pradhan , and J. Liu . 2012. “Coexistence Between Wildlife and Humans at Fine Spatial Scales.” Proceedings of the National Academy of Sciences 109: 15360–15365. 10.1073/pnas.1210490109. PMC3458348 22949642

[ece372106-bib-0010] Dai, J. , J. Chen , Z. Luo , and W. Zhou . 2023. “Coping With Giant Panda Nature Reserve Protection Dilemmas in China: Social Capital's Role in Forest Conservation.” Global Ecology and Conservation 42: e02379. 10.1016/j.gecco.2023.e02379.

[ece372106-bib-0011] Dong, X. , J. Gong , W. Zhang , S. Zhang , Y. Hu , and G. Yang . 2024. “Importance of Including Key Biodiversity Areas in China's Conservation Area‐Based Network.” Biological Conservation 296: 110676. 10.1016/j.biocon.2024.110676.

[ece372106-bib-0012] Dong, X. , J. D. Zhang , X. D. Gu , Y. J. Wang , W. K. Bai , and Q. Y. Huang . 2021. “Evaluating Habitat Suitability and Potential Dispersal Corridors Across the Distribution Landscape of the Chinese Red Panda (*Ailurus Styani*) in Sichuan, China.” Global Ecology and Conservation 28: e01705. 10.1016/j.gecco.2021.e01705.

[ece372106-bib-0013] Elith, J. , C. H. Graham , R. P. Anderson , et al. 2006. “Novel Methods Improve Prediction of Species' Distributions From Occurrence Data.” Ecography 29: 129–151. 10.1111/j.2006.0906-7590.04596.x.

[ece372106-bib-0014] Ferreira, G. B. , L. Thomas , D. J. Ingram , et al. 2023. “Wildlife Response to Management Regime and Habitat Loss in the Terai Arc Landscape of Nepal.” Biological Conservation 288: 110–334. 10.1016/j.biocon.2023.110334.

[ece372106-bib-0015] Fourcade, Y. , J. O. Engler , D. Rödder , and J. Secondi . 2014. “Mapping Species Distributions With MAXENT Using a Geographically Biased Sample of Presence Data: A Performance Assessment of Methods for Correcting Sampling Bias.” PLoS One 9: e97122. 10.1371/journal.pone.0097122. 24818607 PMC4018261

[ece372106-bib-0016] Fu, L. Q. , W. K. Bai , Z. S. Guo , et al. 2020. “Biodiversity and Spatio‐Temporal Patterns of Mammals in the Mabian Dafengding National Nature Reserve Using Camera Traps.” Sichuan Journal of Zoology 39, no. 4: 442–452. 10.11984/j.issn.1000-7083.20190375.

[ece372106-bib-0017] Hull, V. , W. H. Xu , W. Liu , et al. 2011. “Evaluating the Efficacy of Zoning Designations for Protected Area Management.” Biological Conservation 144: 3028–3037. 10.1016/j.biocon.2011.09.007.

[ece372106-bib-0018] Hull, V. , J. Zhang , J. Huang , et al. 2016. “Habitat Use and Selection by Giant Pandas.” PLoS One 11: e0162266. 10.1371/journal.pone.0162266. 27627805 PMC5023135

[ece372106-bib-0019] Hull, V. , J. Zhang , S. Zhou , et al. 2014. “Impact of Livestock on Giant Pandas and Their Habitat.” Journal for Nature Conservation 22: 256–264. 10.1016/j.jnc.2014.02.003.

[ece372106-bib-0020] Ju, X. , B. Wang , L. Wu , X. Zhang , Q. Wu , and G. Han . 2024. “Grazing Decreases Net Ecosystem Carbon Exchange by Decreasing Shrub and Semi‐Shrub Biomass in a Desert Steppe.” Ecology and Evolution 14, no. 6: 1. 10.1002/ece3.11528. PMC11199334 38932943

[ece372106-bib-0021] Kang, D. , L. Zhao , and G. Song . 2011. “Competition Relationship Between Giant Panda and Livestock in Wanglang National Nature Reserve, Sichuan.” Journal of Northeast Forestry University 39: 74–76. 10.13759/j.cnki.dlxb.2011.07.001.

[ece372106-bib-0022] Kramer‐Schadt, S. , J. Niedballa , J. D. Pilgrim , et al. 2013. “The Importance of Correcting for Sampling Bias in MaxEnt Species Distribution Models.” Diversity and Distributions 19: 1366–1379. 10.1111/ddi.12096.

[ece372106-bib-0023] Lama, S. , S. Shrestha , N. P. Koju , A. P. Sherpa , and M. Tamang . 2020. “Assessment of the Impacts of Livestock Grazing on Endangered Red Panda (*Ailurus fulgens*) Habitat in Eastern Nepal.” Open Journal of Ecology 10: 97–110. 10.4236/oje.2020.103008.

[ece372106-bib-0024] Li, C. , T. Connor , W. K. Bai , et al. 2019. “Dynamics of the Giant Panda Habitat Suitability in Response to Changing Anthropogenic Disturbance in the Liangshan Mountains.” Biological Conservation 237: 445–455. 10.1016/j.biocon.2019.07.018.

[ece372106-bib-0025] Limbu, A. , A. Thapa , L. Khanal , S. Gurung , N. J. Cruz , and T. B. Thapa . 2024. “Habitat Characteristics of the Endangered Himalayan Red Panda in Panchthar‐Ilam‐Taplejung Corridor, Eastern Nepal.” Ecologies 5, no. 3: 342–353. 10.3390/ecologies5030021.

[ece372106-bib-0026] Liu, W. , X. X. Nie , F. J. Chen , et al. 2024. “Field Survey Data for Conservation: Evaluating Suitable Habitat of Chinese Pangolin at the County‐Level in Eastern China (2000–2040).” Ecology and Evolution 14, no. 6: e11512. 10.1002/ece3.11512. 38835522 PMC11147814

[ece372106-bib-0028] Llorente‐Culebras, S. , R. Ladle , and A. M. C. Santos . 2023. “Publication Trends in Global Biodiversity Research on Protected Areas.” Biological Conservation 28: 109988. 10.1016/j.biocon.2023.109988.

[ece372106-bib-0029] Mao, Z. E. , Y. Hong , Y. J. Wang , et al. 2023. “Dynamic Patterns of Habitat Connectivity of Local Giant Panda Populaions in Liangshan Mountains.” Chisene Journal Ecology 42, no. 3: 708–715. 10.13292/j.1000-4890.202303.016.

[ece372106-bib-0030] Martineau, J. , D. Pothier , and D. Fortin . 2016. “Processes Driving Short‐Term Temporal Dynamics of Small Mammal Distribution in Human‐Disturbed Environments.” Oecologia 181, no. 3: 831–840. 10.1007/s00442-016-3613-6. 27003700

[ece372106-bib-0032] Ning, H. , D. Qiang , J. Ran , Y. Jiao , C. Yong , and Z. Cheng . 2014. “A Corridor Design for the Giant Panda in the Niba Mountain of China.” Chinese Journal of Applied & Environmental Biology 20: 1039–1045. 10.3724/SP.J.1145.2014.06003.

[ece372106-bib-0033] Pema, D. , H. Tatyana , B. Damber , P. Ugyen , L. Choki , and G. Jigme . 2020. “Habitat Requirements of the Himalayan Red Panda (*Ailurus fulgens*) and Threat Analysis in Jigme Dorji National Park, Bhutan.” Ecology and Evolution 10: 9444–9453. 10.1002/ece3.6632. 32953073 PMC7487235

[ece372106-bib-0034] Phillips, S. J. , R. P. Anderson , and R. E. Schapire . 2006. “Maximum Entropy Modeling of Species Geographic Distributions.” Ecological Modelling 190: 231–259. 10.1016/j.ecolmodel.2005.03.026.

[ece372106-bib-0035] Powell, R. A. 2000. “Animal Home Ranges and Territories and Home Range Estimators.” In Research Techniques in Animal Ecology: Controversies and Consequences, vol. 442, 65–110. Columbia University Press. https://www.researchgate.net/publication/236982035.

[ece372106-bib-0036] Ran, J. , S. Liu , H. Wang , Z. Sun , Z. Zeng , and S. Liu . 2003. “Habitat Selection by Giant Pandas and Grazing Livestock in the Xiaoxiangling Mountains of Sichuan Province.” Acta Ecologica Sinica 23: 2253–2259. 10.3321/j.issn:1000-0933.2003.11.007.

[ece372106-bib-0037] Ruan, T. , W. Wei , Z. J. Zhang , and H. Zhou . 2024. “Research on the Changes in Distribution and Habitat Suitability of the Chinese Red Panda Population.” Animals 14, no. 3: 2076–2615. 10.3390/ani14030424. 38338067 PMC10854785

[ece372106-bib-0038] Shah, M. , C. J. Shah , A. Mevada , et al. 2020. “Wildlife Conservation Strategies and Management in India: An Overview.” International Journal of Scientific Research in Science and Technology 6: 108–112. 10.32628/ijsrst196528.

[ece372106-bib-0039] Sikha, K. , M. Tek , M. Brendan , et al. 2021. “Reaching Over the Gap: A Review of Trends in and Status of Red Panda Research Over 193 Years (1827–2020).” Science of the Total Environment 781: 1–12. 10.1016/j.scitotenv.2021.146659. 33794452

[ece372106-bib-0040] State Forestry Administration . 2015. “Results of the Fourth National Survey on the Giant Panda.”

[ece372106-bib-0041] Tang, J. F. , J. Zhang , X. Z. Zhao , et al. 2022. “The Fate of Giant Panda and Its Sympatric Mammals Under Future Climate Change.” Biological Conservation 274: 109715. 10.1016/j.biocon.2022.109715.

[ece372106-bib-0042] Thakur, M. L. , L. Jain , V. Negi , S. K. Narang , and J. Singh . 2024. “Status and Phylogeny of Threatened Wildlife Species Found in Himachal Pradesh, India.” Proceedings of the Zoological Society 77, no. 2: 272–279. 10.1007/s12595-024-00532-6.

[ece372106-bib-0043] Thapa, A. , Y. B. Hu , P. Chandra , et al. 2020. “The Endangered Red Panda in Himalayas: Potential Distribution and Ecological Habitat Associates.” Global Ecology and Conservation 21: e00890. 10.1016/j.gecco.2019.e00890.

[ece372106-bib-0044] Thapa, A. , Y. B. Hu , and F. W. Wei . 2018. “The Endangered Red Panda (*Ailurus fulgens*): Ecology and Conservation Approaches Across the Entire Range.” Biology Conservation 220: 112–121. 10.1016/j.biocon.2018.02.014.

[ece372106-bib-0045] Viña, A. , X. Chen , W. J. Mcconnell , et al. 2011. “Effects of Natural Disasters on Conservation Policies: The Case of the 2008 Wenchuan Earthquake, China.” Ambio 40: 274–284. 10.2307/41417278. 21644456 PMC3357801

[ece372106-bib-0046] Wang, J. H. , and S. Y. Li . 2024. “Pathways to Consolidate Poverty Alleviation Achievements and Enable Effective Rural Revitalization: Empirical Evidence From Liangshan Yi Autonomous Prefecture, China.” Chinese Rural Economy 3, no. 3: 58–73. 10.13872/j.1002-8870.2024.03.005.

[ece372106-bib-0047] Wei, F. W. , R. Swaisgood , Y. B. Hu , et al. 2015. “Progress in the Ecology and Conservation of Giant Pandas.” Conservation Biology 29, no. 6: 1497–1507. 10.1111/cobi.12582. 26372302

[ece372106-bib-0048] Wei, W. , R. R. Swaisgood , Q. Dai , et al. 2018. “Giant Panda Distributional and Habitat‐Use Shifts in a Changing Landscape.” Conservation Letters 11, no. 6: e12575. 10.1111/conl.12575.

[ece372106-bib-0049] Western, D. , S. Russell , and I. Cuthill . 2009. “The Status of Wildlife in Protected Areas Compared to Non‐Protected Areas of Kenya.” PLoS One 4: e6140. 10.1371/journal.pone.0006140. 19584912 PMC2702096

[ece372106-bib-0050] White, A. M. , T. G. Holland , E. S. Abelson , A. C. Kretchun , J. Maxwell , and R. M. Scheller . 2022. “Simulating Wildlife Habitatdynamics Over the Next Century to Help Inform Best Management Strategies for Biodiversity in the Lake Tahoe Basin.” California. Ecology and Society 27, no. 2: 31. 10.5751/ES-13301-270231.

[ece372106-bib-0051] Xiang, Q. , H. Yu , H. Huang , et al. 2024. “The Impact of Grazing Activities and Environmental Conditions on the Stability of Alpine Grassland Ecosystems.” Journal of Environmental Management 360: 121176. 10.1016/j.jenvman.2024.121176. 38759547

[ece372106-bib-0052] Yang, H. , A. Viña , Y. Tang , et al. 2017. “Range‐Wide Evaluation of Wildlife Habitat Change: A Demonstration Using Giant Pandas.” Biological Conservation 213: 203–209. 10.1016/j.biocon.2017.07.010.

[ece372106-bib-0053] Yang, Z. S. , X. D. Gu , Y. G. Nie , et al. 2018. “Reintroduction of the Giant Panda Into the Wild: A Good Start Suggests a Bright Future.” Biological Conservation 217: 181–186. 10.1016/j.biocon.2017.08.012.

[ece372106-bib-0054] Ying, Z. , H. Han , Y. H. Gong , et al. 2023. “Feeding Habits and Foraging Patch Selection Strategy of the Giant Panda in the Meigu Dafengding National Nature Reserve, Sichuan Province, China.” Environmental Science and Pollution Research 30, no. 17: 49125–49135. 10.1007/s11356-023-25769-0. 36773257

[ece372106-bib-0055] Zang, Z. H. , G. Z. Shen , G. F. Ren , et al. 2017. “Thermal Habitat of Giant Panda Has Shrunk by Climate Warming Over the Past Half Century.” Biological Conservation 211: 125–133. 10.1016/j.ecolind.2022.109452.

[ece372106-bib-0056] Zhang, J. , V. Hull , and Z. Ouyang . 2013. “A Review of Home Range Studies.” Acta Ecologica Sinica 33: 3269–3279. 10.1093/jmammal/gyv118.

[ece372106-bib-0057] Zhang, J. D. , V. Hull , J. Y. Huang , et al. 2015. “Activity Patterns of the Giant Panda (*Ailuropoda melanoleuca*).” Journal of Mammalogy 96, no. 6: 1116–1127. 10.1093/jmammal/gyv118.

[ece372106-bib-0058] Zhang, J. D. , V. Hull , Z. Y. Ouyang , et al. 2017. “Divergent Responses of Sympatric Species to Livestock Encroachment at Fine Spatiotemporal Scales.” Biological Conservation 209: 119–129. 10.1016/j.biocon.2017.02.014.

[ece372106-bib-0059] Zhang, J. J. , S. Pan , Q. B. Che , W. Wei , X. Z. Zhao , and J. F. Tang . 2022. “Impacts of Climate Change on the Distributions and Diversity of the Giant Panda With Its Sympatric Mammalian Species.” Ecological Indicators 144: 109452. 10.1016/j.biocon.2017.05.011.

[ece372106-bib-0060] Zhang, Z. J. , J. C. Hu , and Z. X. Hang . 2011. “Activity Patterns of Wild Red Panda in Fengtongzhai Nature Reserve, China.” Italian Journal of Zoology 78, no. 3: 398–404. 10.1080/11250003.2011.563248.

[ece372106-bib-0061] Zhang, Z. J. , Y. X. Li , W. Wei , H. Zhou , and M. S. Hong . 2017. “On Nutrition Ecology of Wild Red Panda: Research Status and Perspective.” Journal of China West Normal Uneversity (Natural Sciences) 38, no. 1: 1–5.

[ece372106-bib-0062] Zhang, Z. J. , F. W. Wei , M. Li , and J. C. Hu . 2006. “Winter Microhabitat Separation Between Giant and Red Pandas in Bashania Faberi Bamboo Forest in Fengtongzhai Nature Reserve.” Journal of Wildlife Management 70: 231–235. 10.2307/3803565.

[ece372106-bib-0063] Zhou, W. , B. Zheng , Z. Q. Zhang , Z. J. Song , and W. Duan . 2021. “The Role of Eco‐Tourism in Ecological Conservation in Giant Panda Nature Reserve.” Journal of Environmental Management 295: 113077. 10.1016/j.jenvman.2021.113077. 34146778

