# Supplementary material for: Livestock Grazing Management Influences Survival of Giant and Red Pandas in Southwest China
Source: Ecol Evol. 2025 Sep 18;15(9):e72106. doi: 10.1002/ece3.72106 (PMC12445196; doi:10.1002/ece3.72106)
Supplement: Supplementary file 1 — Data S1: ece372106‐sup‐0001‐TableS1‐S5.docx. [file ECE3-15-e72106-s001.docx]

Supplementary table 1. Location data of giant pandas, red pandas and livestock under the different grazing conditions in Meigu Dafengding National Nature Reserve, China.

| Giant pandas | | Red pandas | | Livestock | |
| --- | --- | --- | --- | --- | --- |
| X | Y | X | Y | X | Y |
| 102.90054 | 28.61666 | 102.90518 | 28.65470 | 102.89327 | 28.61263 |
| 102.89458 | 28.61289 | 102.96602 | 28.60706 | 102.89990 | 28.62320 |
| 102.89531 | 28.61427 | 102.92371 | 28.56915 | 102.94496 | 28.63303 |
| 102.89690 | 28.61694 | 102.91249 | 28.58780 | 102.95500 | 28.64154 |
| 102.89804 | 28.61789 | 103.00050 | 28.66292 | 102.95179 | 28.63987 |
| 102.89793 | 28.62115 | 102.99970 | 28.66177 | 102.94811 | 28.63616 |
| 102.89536 | 28.62530 | 103.01854 | 28.66746 | 102.91172 | 28.64479 |
| 102.89784 | 28.62576 | 102.91299 | 28.61379 | 102.90689 | 28.63625 |
| 102.90165 | 28.62583 | 102.94228 | 28.62073 | 102.88888 | 28.62999 |
| 102.93500 | 28.63275 | 102.97652 | 28.64097 | 102.95755 | 28.65284 |
| 102.93763 | 28.63546 | 102.96959 | 28.58938 | 102.97182 | 28.64968 |
| 102.93896 | 28.63264 | 102.96749 | 28.59720 | 102.94136 | 28.61272 |
| 102.94693 | 28.63592 | 103.21824 | 28.65390 | 102.92746 | 28.61009 |
| 102.94492 | 28.64920 | 102.98741 | 28.60882 | 102.93131 | 28.63235 |
| 102.94690 | 28.64546 | 103.15584 | 28.66568 | 102.93134 | 28.56995 |
| 102.94702 | 28.64456 | 103.15862 | 28.66736 | 102.96806 | 28.59324 |
| 102.95475 | 28.63848 | 103.20333 | 28.65455 | 102.96325 | 28.59049 |
| 102.95336 | 28.63867 | 103.02446 | 28.63978 | 102.91574 | 28.60740 |
| 102.95506 | 28.64158 | 103.01649 | 28.66587 | 102.99755 | 28.65818 |
| 102.95183 | 28.63981 | 103.02482 | 28.66314 | 102.99705 | 28.65180 |
| 102.94811 | 28.63616 | 103.03750 | 28.64318 | 102.99653 | 28.64761 |
| 102.96797 | 28.63671 | 103.29120 | 28.70721 | 102.99257 | 28.64520 |
| 102.96896 | 28.63465 | 103.27774 | 28.70772 | 102.96561 | 28.66290 |
| 102.96840 | 28.63161 | 103.12637 | 28.69102 | 102.98207 | 28.65537 |
| 102.91261 | 28.64310 | 103.13386 | 28.69888 | 102.98399 | 28.66264 |
| 102.90902 | 28.64591 | 103.09133 | 28.70275 | 102.98425 | 28.66082 |
| 102.90583 | 28.64866 | 103.09756 | 28.74193 | 102.93490 | 28.62583 |
| 102.90388 | 28.64929 | 103.09991 | 28.73596 | 102.93562 | 28.62372 |
| 102.89935 | 28.65021 | 103.13618 | 28.80577 | 102.92221 | 28.62285 |
| 102.89545 | 28.64958 | 103.16904 | 28.82013 | 102.98485 | 28.61672 |
| 102.89066 | 28.65109 | 103.16804 | 28.81924 | 103.30942 | 28.70315 |
| 102.91177 | 28.65777 | 103.27678 | 28.71452 | 103.23285 | 28.66172 |
| 102.91220 | 28.65617 | 103.27162 | 28.72328 | 103.24051 | 28.67982 |
| 102.90706 | 28.65584 | 103.23657 | 28.70397 | 103.24659 | 28.67644 |
| 102.90158 | 28.65591 | 103.23079 | 28.69160 | 103.22044 | 28.65671 |
| 102.89966 | 28.66062 | 103.18390 | 28.67723 | 103.09627 | 28.69205 |
| 102.89754 | 28.66336 | 103.30378 | 28.72056 | 103.09649 | 28.70263 |
| 102.91763 | 28.63382 | 103.30338 | 28.71916 | 103.10111 | 28.69762 |
| 102.91388 | 28.63618 | 103.29846 | 28.71920 | 103.10047 | 28.73352 |
| 102.90858 | 28.63635 | 103.28573 | 28.71546 | 103.08189 | 28.72943 |
| 102.90484 | 28.63646 | 103.32962 | 28.72800 | 103.08784 | 28.71935 |
| 102.90302 | 28.63703 | 103.28183 | 28.79411 | 103.08401 | 28.74225 |
| 102.89988 | 28.63515 | 103.24013 | 28.80825 |  |  |
| 102.89437 | 28.63301 | 103.22526 | 28.80273 |  |  |
| 102.89043 | 28.63045 |  |  |  |  |
| 102.88800 | 28.63038 |  |  |  |  |
| 102.88973 | 28.63010 |  |  |  |  |
| 102.97120 | 28.62368 |  |  |  |  |
| 102.97573 | 28.62156 |  |  |  |  |
| 102.97629 | 28.62225 |  |  |  |  |
| 102.97716 | 28.62316 |  |  |  |  |
| 102.98027 | 28.62528 |  |  |  |  |
| 102.98144 | 28.62736 |  |  |  |  |
| 102.95892 | 28.64406 |  |  |  |  |
| 102.95863 | 28.64819 |  |  |  |  |
| 102.95757 | 28.65155 |  |  |  |  |
| 102.96109 | 28.65528 |  |  |  |  |
| 102.96379 | 28.65567 |  |  |  |  |
| 102.96891 | 28.65605 |  |  |  |  |
| 102.97201 | 28.65469 |  |  |  |  |
| 102.97430 | 28.65199 |  |  |  |  |
| 102.96941 | 28.64669 |  |  |  |  |
| 102.96283 | 28.64383 |  |  |  |  |
| 102.93999 | 28.61634 |  |  |  |  |
| 102.94135 | 28.61265 |  |  |  |  |
| 102.93720 | 28.61123 |  |  |  |  |
| 102.93453 | 28.60993 |  |  |  |  |
| 102.93286 | 28.60964 |  |  |  |  |
| 102.92719 | 28.61025 |  |  |  |  |
| 102.92661 | 28.61353 |  |  |  |  |
| 102.92402 | 28.61719 |  |  |  |  |
| 102.92128 | 28.63232 |  |  |  |  |
| 102.92817 | 28.63481 |  |  |  |  |
| 102.93048 | 28.63351 |  |  |  |  |
| 102.93310 | 28.63094 |  |  |  |  |
| 102.96153 | 28.60940 |  |  |  |  |
| 102.96156 | 28.60941 |  |  |  |  |
| 102.96874 | 28.60784 |  |  |  |  |
| 102.96661 | 28.60587 |  |  |  |  |
| 102.96489 | 28.60568 |  |  |  |  |
| 102.96322 | 28.60703 |  |  |  |  |
| 102.94927 | 28.62622 |  |  |  |  |
| 102.95325 | 28.62166 |  |  |  |  |
| 102.95098 | 28.62183 |  |  |  |  |
| 102.94947 | 28.62376 |  |  |  |  |
| 102.91859 | 28.56951 |  |  |  |  |
| 102.91946 | 28.56908 |  |  |  |  |
| 102.92234 | 28.56834 |  |  |  |  |
| 102.92569 | 28.57047 |  |  |  |  |
| 102.93142 | 28.56996 |  |  |  |  |
| 102.96576 | 28.59038 |  |  |  |  |
| 102.97124 | 28.59206 |  |  |  |  |
| 102.96953 | 28.59493 |  |  |  |  |
| 102.96760 | 28.59292 |  |  |  |  |
| 102.96578 | 28.59484 |  |  |  |  |
| 102.96540 | 28.59288 |  |  |  |  |
| 102.96530 | 28.59203 |  |  |  |  |
| 102.96322 | 28.59047 |  |  |  |  |
| 102.90911 | 28.60076 |  |  |  |  |
| 102.90914 | 28.60156 |  |  |  |  |
| 102.90944 | 28.60315 |  |  |  |  |
| 102.91110 | 28.60469 |  |  |  |  |
| 102.91058 | 28.60495 |  |  |  |  |
| 102.91239 | 28.60496 |  |  |  |  |
| 102.91472 | 28.60645 |  |  |  |  |
| 102.91667 | 28.60807 |  |  |  |  |
| 102.91905 | 28.60899 |  |  |  |  |
| 102.95044 | 28.60284 |  |  |  |  |
| 102.95662 | 28.61218 |  |  |  |  |
| 102.95150 | 28.60738 |  |  |  |  |
| 102.95631 | 28.60689 |  |  |  |  |
| 102.95731 | 28.60602 |  |  |  |  |
| 102.95546 | 28.60316 |  |  |  |  |
| 102.95456 | 28.59949 |  |  |  |  |
| 102.95010 | 28.59937 |  |  |  |  |
| 102.91229 | 28.58770 |  |  |  |  |
| 102.91586 | 28.58898 |  |  |  |  |
| 102.91926 | 28.59005 |  |  |  |  |
| 102.92038 | 28.59079 |  |  |  |  |
| 102.92547 | 28.59002 |  |  |  |  |
| 102.92936 | 28.59172 |  |  |  |  |
| 103.00134 | 28.66321 |  |  |  |  |
| 102.99970 | 28.66175 |  |  |  |  |
| 102.99689 | 28.65749 |  |  |  |  |
| 102.99563 | 28.65603 |  |  |  |  |
| 102.99711 | 28.65167 |  |  |  |  |
| 102.99652 | 28.64762 |  |  |  |  |
| 102.99265 | 28.64500 |  |  |  |  |
| 102.95402 | 28.65823 |  |  |  |  |
| 102.95416 | 28.65753 |  |  |  |  |
| 102.95566 | 28.65647 |  |  |  |  |
| 102.96107 | 28.65731 |  |  |  |  |
| 102.96878 | 28.66537 |  |  |  |  |
| 102.96591 | 28.66321 |  |  |  |  |
| 102.96362 | 28.66070 |  |  |  |  |
| 102.96168 | 28.65774 |  |  |  |  |
| 102.98986 | 28.65012 |  |  |  |  |
| 102.98374 | 28.64799 |  |  |  |  |
| 102.98254 | 28.65102 |  |  |  |  |
| 102.98213 | 28.65538 |  |  |  |  |
| 102.98458 | 28.66646 |  |  |  |  |
| 102.98400 | 28.66265 |  |  |  |  |
| 102.98569 | 28.65822 |  |  |  |  |
| 102.91254 | 28.62237 |  |  |  |  |
| 102.91086 | 28.61854 |  |  |  |  |
| 102.91781 | 28.62238 |  |  |  |  |
| 102.91901 | 28.62423 |  |  |  |  |
| 102.93491 | 28.62582 |  |  |  |  |
| 102.93581 | 28.62335 |  |  |  |  |
| 102.93749 | 28.62130 |  |  |  |  |
| 102.93692 | 28.61918 |  |  |  |  |
| 102.92416 | 28.61798 |  |  |  |  |
| 102.92393 | 28.62002 |  |  |  |  |
| 102.92493 | 28.62221 |  |  |  |  |
| 102.92196 | 28.62357 |  |  |  |  |
| 102.92148 | 28.62726 |  |  |  |  |
| 103.00373 | 28.63423 |  |  |  |  |
| 103.00070 | 28.63776 |  |  |  |  |
| 102.99666 | 28.64116 |  |  |  |  |
| 102.99275 | 28.63649 |  |  |  |  |
| 102.99129 | 28.63172 |  |  |  |  |
| 102.99232 | 28.62962 |  |  |  |  |
| 102.99302 | 28.62696 |  |  |  |  |
| 102.99422 | 28.62159 |  |  |  |  |
| 103.03385 | 28.62096 |  |  |  |  |
| 103.03174 | 28.62285 |  |  |  |  |
| 103.02734 | 28.62675 |  |  |  |  |
| 103.02217 | 28.62739 |  |  |  |  |
| 103.01864 | 28.62888 |  |  |  |  |
| 103.01614 | 28.62913 |  |  |  |  |
| 103.01487 | 28.62893 |  |  |  |  |
| 103.00811 | 28.63068 |  |  |  |  |
| 102.98425 | 28.61090 |  |  |  |  |
| 102.98443 | 28.61213 |  |  |  |  |
| 102.98441 | 28.61599 |  |  |  |  |
| 102.98561 | 28.61789 |  |  |  |  |
| 103.25875 | 28.68897 |  |  |  |  |
| 103.25603 | 28.69619 |  |  |  |  |
| 103.25606 | 28.68979 |  |  |  |  |
| 103.25650 | 28.68691 |  |  |  |  |
| 103.28723 | 28.70439 |  |  |  |  |
| 103.28613 | 28.70447 |  |  |  |  |
| 103.28537 | 28.70100 |  |  |  |  |
| 103.28707 | 28.69935 |  |  |  |  |
| 103.32189 | 28.71297 |  |  |  |  |
| 103.32079 | 28.71107 |  |  |  |  |
| 103.31872 | 28.70883 |  |  |  |  |
| 103.31568 | 28.70721 |  |  |  |  |
| 103.31218 | 28.70437 |  |  |  |  |
| 103.30942 | 28.70315 |  |  |  |  |
| 103.22142 | 28.66862 |  |  |  |  |
| 103.22260 | 28.66802 |  |  |  |  |
| 103.22707 | 28.66643 |  |  |  |  |
| 103.23006 | 28.66313 |  |  |  |  |
| 103.23314 | 28.66089 |  |  |  |  |
| 103.24939 | 28.67988 |  |  |  |  |
| 103.24772 | 28.68170 |  |  |  |  |
| 103.24411 | 28.68058 |  |  |  |  |
| 103.24139 | 28.67953 |  |  |  |  |
| 103.24608 | 28.67675 |  |  |  |  |
| 103.25028 | 28.67594 |  |  |  |  |
| 103.22044 | 28.65671 |  |  |  |  |
| 103.22044 | 28.65671 |  |  |  |  |
| 103.22044 | 28.65671 |  |  |  |  |
| 103.22050 | 28.65669 |  |  |  |  |
| 103.27727 | 28.72169 |  |  |  |  |
| 103.27838 | 28.71659 |  |  |  |  |
| 103.27774 | 28.71282 |  |  |  |  |
| 103.27810 | 28.70850 |  |  |  |  |
| 103.27524 | 28.70523 |  |  |  |  |
| 103.27467 | 28.70088 |  |  |  |  |
| 103.01574 | 28.64643 |  |  |  |  |
| 103.01524 | 28.64810 |  |  |  |  |
| 103.01538 | 28.65118 |  |  |  |  |
| 103.01696 | 28.64998 |  |  |  |  |
| 103.01789 | 28.64759 |  |  |  |  |
| 103.01950 | 28.64568 |  |  |  |  |
| 102.98952 | 28.57058 |  |  |  |  |
| 102.99207 | 28.57062 |  |  |  |  |
| 103.03360 | 28.63474 |  |  |  |  |
| 103.02963 | 28.63308 |  |  |  |  |
| 103.02812 | 28.63638 |  |  |  |  |
| 103.02194 | 28.64102 |  |  |  |  |
| 103.01790 | 28.64147 |  |  |  |  |
| 103.01069 | 28.64245 |  |  |  |  |
| 103.01069 | 28.63699 |  |  |  |  |
| 103.01837 | 28.63537 |  |  |  |  |
| 103.01881 | 28.66413 |  |  |  |  |
| 103.01562 | 28.66545 |  |  |  |  |
| 103.01725 | 28.66657 |  |  |  |  |
| 103.01893 | 28.66873 |  |  |  |  |
| 103.02024 | 28.66832 |  |  |  |  |
| 103.02118 | 28.66584 |  |  |  |  |
| 103.02513 | 28.66048 |  |  |  |  |
| 103.03115 | 28.65412 |  |  |  |  |
| 103.02599 | 28.65156 |  |  |  |  |
| 103.02474 | 28.65150 |  |  |  |  |
| 103.02271 | 28.64691 |  |  |  |  |
| 103.02173 | 28.64548 |  |  |  |  |
| 102.98752 | 28.58759 |  |  |  |  |
| 102.98858 | 28.58673 |  |  |  |  |
| 102.99066 | 28.58701 |  |  |  |  |
| 102.99177 | 28.58516 |  |  |  |  |
| 103.06077 | 28.65885 |  |  |  |  |
| 103.05175 | 28.66598 |  |  |  |  |
| 103.04853 | 28.67032 |  |  |  |  |
| 103.05779 | 28.66809 |  |  |  |  |
| 103.05093 | 28.63691 |  |  |  |  |
| 103.04302 | 28.64367 |  |  |  |  |
| 103.04080 | 28.63930 |  |  |  |  |
| 103.04361 | 28.63813 |  |  |  |  |
| 103.04792 | 28.63753 |  |  |  |  |
| 103.04922 | 28.64294 |  |  |  |  |
| 103.05125 | 28.64475 |  |  |  |  |
| 103.08067 | 28.66018 |  |  |  |  |
| 103.08036 | 28.66341 |  |  |  |  |
| 103.07877 | 28.66523 |  |  |  |  |
| 103.07759 | 28.66633 |  |  |  |  |
| 103.07532 | 28.66598 |  |  |  |  |
| 103.07321 | 28.66656 |  |  |  |  |
| 103.07033 | 28.66596 |  |  |  |  |
| 103.06870 | 28.66251 |  |  |  |  |
| 103.07564 | 28.63880 |  |  |  |  |
| 103.07204 | 28.64248 |  |  |  |  |
| 103.06902 | 28.64293 |  |  |  |  |
| 103.06803 | 28.64502 |  |  |  |  |
| 103.06738 | 28.64530 |  |  |  |  |
| 103.06495 | 28.64865 |  |  |  |  |
| 103.06601 | 28.65164 |  |  |  |  |
| 103.06569 | 28.65432 |  |  |  |  |
| 103.06321 | 28.65540 |  |  |  |  |
| 103.03634 | 28.65743 |  |  |  |  |
| 103.03647 | 28.66154 |  |  |  |  |
| 103.03431 | 28.66600 |  |  |  |  |
| 103.04090 | 28.66733 |  |  |  |  |
| 103.04351 | 28.67001 |  |  |  |  |
| 103.04357 | 28.64609 |  |  |  |  |
| 103.04040 | 28.64962 |  |  |  |  |
| 103.03444 | 28.65149 |  |  |  |  |
| 103.03393 | 28.65492 |  |  |  |  |
| 103.06242 | 28.70244 |  |  |  |  |
| 103.06719 | 28.70193 |  |  |  |  |
| 103.06766 | 28.69847 |  |  |  |  |
| 103.06957 | 28.69717 |  |  |  |  |
| 103.06760 | 28.69322 |  |  |  |  |
| 103.06484 | 28.69031 |  |  |  |  |
| 103.06319 | 28.68686 |  |  |  |  |
| 103.06254 | 28.68396 |  |  |  |  |
| 103.01865 | 28.67433 |  |  |  |  |
| 103.00799 | 28.67646 |  |  |  |  |
| 103.00392 | 28.67790 |  |  |  |  |
| 103.00117 | 28.67758 |  |  |  |  |
| 103.00399 | 28.67447 |  |  |  |  |
| 103.00799 | 28.67059 |  |  |  |  |
| 103.01558 | 28.67093 |  |  |  |  |
| 103.08458 | 28.69555 |  |  |  |  |
| 103.08606 | 28.69132 |  |  |  |  |
| 103.08760 | 28.68839 |  |  |  |  |
| 103.09131 | 28.68928 |  |  |  |  |
| 103.08821 | 28.68450 |  |  |  |  |
| 103.09058 | 28.68751 |  |  |  |  |
| 103.09338 | 28.69084 |  |  |  |  |
| 103.09876 | 28.69076 |  |  |  |  |
| 103.10386 | 28.69208 |  |  |  |  |
| 103.10116 | 28.69045 |  |  |  |  |
| 103.09367 | 28.68901 |  |  |  |  |
| 103.09088 | 28.70163 |  |  |  |  |
| 103.09089 | 28.70259 |  |  |  |  |
| 103.09165 | 28.70441 |  |  |  |  |
| 103.09373 | 28.70385 |  |  |  |  |
| 103.09657 | 28.70237 |  |  |  |  |
| 103.09886 | 28.70076 |  |  |  |  |
| 103.10045 | 28.69904 |  |  |  |  |
| 103.10127 | 28.69745 |  |  |  |  |
| 103.09822 | 28.74064 |  |  |  |  |
| 103.09871 | 28.73970 |  |  |  |  |
| 103.09966 | 28.73753 |  |  |  |  |
| 103.10067 | 28.73532 |  |  |  |  |
| 103.10080 | 28.73496 |  |  |  |  |
| 103.10212 | 28.73181 |  |  |  |  |
| 103.10371 | 28.72953 |  |  |  |  |
| 103.10428 | 28.72621 |  |  |  |  |
| 103.10386 | 28.72253 |  |  |  |  |
| 103.08312 | 28.73451 |  |  |  |  |
| 103.08240 | 28.73255 |  |  |  |  |
| 103.08308 | 28.72570 |  |  |  |  |
| 103.08584 | 28.72275 |  |  |  |  |
| 103.08676 | 28.72159 |  |  |  |  |
| 103.08399 | 28.71751 |  |  |  |  |
| 103.08811 | 28.71863 |  |  |  |  |
| 103.09018 | 28.71636 |  |  |  |  |
| 103.12045 | 28.70624 |  |  |  |  |
| 103.12015 | 28.69805 |  |  |  |  |
| 103.12960 | 28.70015 |  |  |  |  |
| 103.13039 | 28.69735 |  |  |  |  |
| 103.12788 | 28.69590 |  |  |  |  |
| 103.12577 | 28.69091 |  |  |  |  |
| 103.12542 | 28.68729 |  |  |  |  |
| 103.12417 | 28.68461 |  |  |  |  |
| 103.08032 | 28.75306 |  |  |  |  |
| 103.08756 | 28.75372 |  |  |  |  |
| 103.08860 | 28.74904 |  |  |  |  |
| 103.08666 | 28.74583 |  |  |  |  |
| 103.07966 | 28.74293 |  |  |  |  |
| 103.08368 | 28.73656 |  |  |  |  |

Supplementary table 2. Environmental variables for assessing the preferred habitat of the giant and red pandas.

| Environment variables | Description | Unit |
| --- | --- | --- |
| Altitude | The altitude of the animals' locations | m |
| Slope | The slope of the animals' locations | ° |
| Aspect | The aspect of the animals' locations | ° |
| Grazing | The distance from the location of livestock | m |
| Residents | The distance from the residents | m |
| River | The distance from the river | m |
| Road | The distance from the road | m |
| BIO1 | Annual average temperature | ℃ |
| BIO2 | Average of the daily range of monthly mean temperature | ℃ |
| BIO3 | Isothermality |  |
| BIO4 | Temperature seasonality (standard deviation *100) | ℃ |
| BIO5 | The highest temperature in the hottest month | ℃ |
| BIO6 | The lowest temperature in the coldest month | ℃ |
| BIO7 | Temperature annual range | ℃ |
| BIO8 | The average temperature of the quarter with the highest precipitation | ℃ |
| BIO9 | Mean temperature of the driest quarter | ℃ |
| BIO10 | The average temperature of the quarter with the highest temperature | ℃ |
| BIO11 | The average temperature of the quarter with the lowest temperature | ℃ |
| BIO12 | Annual total precipitation | mm |
| BIO13 | The precipitation in the month with the highest precipitation | mm |
| BIO14 | The precipitation in the month with the lowest precipitation | mm |
| BIO15 | The standard deviation of monthly precipitation / the average monthly precipitation × 100 |  |
| BIO16 | The total precipitation in the quarter with the highest precipitation | mm |
| BIO17 | The total precipitation of the quarter with the lowest precipitation | mm |
| BIO18 | The total precipitation in the quarter with the highest temperature | mm |
| BIO19 | The total precipitation in the quarter with the lowest temperature | mm |

Supplementary table 3. Correlation analysis of environmental variables for the Maxent model.

|  | Bio1 | Bio2 | Bio3 | Bio4 | Bio5 | Bio6 | Bio7 | Bio8 | Bio9 | Bio10 | Bio11 | Bio12 | Bio13 | Bio14 | Bio15 | Bio16 | Bio17 | Bio18 | Bio19 | DEM | Grazing | Residents | river | road | slope | vegetation |
| --- | --- | --- | --- | --- | --- | --- | --- | --- | --- | --- | --- | --- | --- | --- | --- | --- | --- | --- | --- | --- | --- | --- | --- | --- | --- | --- |
| aspect | 0.00 | -0.09 | -0.08 | 0.09 | 0.02 | 0.00 | 0.05 | 0.02 | -0.02 | 0.02 | -0.02 | -0.12 | -0.14 | 0.00 | -0.07 | -0.13 | 0.00 | -0.13 | 0.00 | -0.02 | -0.03 | -0.01 | 0.05 | 0.08 | -0.09 | -0.07 |
| Bio1 |  | -0.49 | -0.65 | 0.72 | 0.99 | 0.99 | 0.76 | 0.99 | 0.98 | 0.99 | 0.98 | 0.23 | -0.32 | 0.82 | -0.55 | 0.31 | 0.82 | 0.31 | 0.82 | -0.97 | 0.31 | -0.34 | -0.13 | -0.09 | 0.09 | 0.58 |
| Bio2 |  |  | 0.95 | -0.83 | -0.54 | -0.49 | -0.55 | -0.57 | -0.34 | -0.57 | -0.34 | 0.18 | 0.66 | -0.58 | 0.73 | 0.35 | -0.60 | 0.35 | -0.60 | 0.60 | -0.19 | -0.13 | 0.01 | -0.36 | 0.07 | -0.26 |
| Bio3 |  |  |  | -0.96 | -0.71 | -0.63 | -0.78 | -0.73 | -0.49 | -0.73 | -0.49 | 0.15 | 0.74 | -0.74 | 0.85 | 0.32 | -0.75 | 0.32 | -0.75 | 0.75 | -0.27 | 0.00 | -0.02 | -0.34 | 0.07 | -0.36 |
| Bio4 |  |  |  |  | 0.79 | 0.68 | 0.92 | 0.80 | 0.57 | 0.80 | 0.57 | -0.12 | -0.76 | 0.80 | -0.88 | -0.27 | 0.82 | -0.27 | 0.82 | -0.81 | 0.30 | -0.11 | 0.03 | 0.28 | -0.07 | 0.42 |
| Bio5 |  |  |  |  |  | 0.98 | 0.83 | 1.00 | 0.95 | 1.00 | 0.95 | 0.16 | -0.41 | 0.84 | -0.62 | 0.22 | 0.84 | 0.22 | 0.84 | -0.97 | 0.30 | -0.34 | -0.11 | -0.03 | 0.06 | 0.57 |
| Bio6 |  |  |  |  |  |  | 0.69 | 0.98 | 0.98 | 0.98 | 0.98 | 0.21 | -0.28 | 0.77 | -0.49 | 0.33 | 0.78 | 0.33 | 0.78 | -0.95 | 0.26 | -0.34 | -0.17 | -0.11 | 0.09 | 0.57 |
| Bio7 |  |  |  |  |  |  |  | 0.82 | 0.63 | 0.82 | 0.63 | -0.03 | -0.67 | 0.83 | -0.84 | -0.14 | 0.84 | -0.14 | 0.84 | -0.80 | 0.34 | -0.26 | 0.08 | 0.18 | -0.03 | 0.46 |
| Bio8 |  |  |  |  |  |  |  |  | 0.95 | 1.00 | 0.95 | 0.17 | -0.41 | 0.85 | -0.63 | 0.22 | 0.86 | 0.22 | 0.86 | -0.98 | 0.32 | -0.31 | -0.11 | -0.02 | 0.06 | 0.57 |
| Bio9 |  |  |  |  |  |  |  |  |  | 0.95 | 1.00 | 0.30 | -0.16 | 0.73 | -0.39 | 0.45 | 0.74 | 0.45 | 0.74 | -0.91 | 0.28 | -0.37 | -0.17 | -0.19 | 0.13 | 0.56 |
| Bio10 |  |  |  |  |  |  |  |  |  |  | 0.95 | 0.17 | -0.41 | 0.85 | -0.63 | 0.22 | 0.86 | 0.22 | 0.86 | -0.98 | 0.32 | -0.31 | -0.11 | -0.02 | 0.06 | 0.57 |
| Bio11 |  |  |  |  |  |  |  |  |  |  |  | 0.30 | -0.16 | 0.73 | -0.39 | 0.45 | 0.74 | 0.45 | 0.74 | -0.91 | 0.28 | -0.37 | -0.17 | -0.19 | 0.13 | 0.56 |
| Bio12 |  |  |  |  |  |  |  |  |  |  |  |  | 0.62 | 0.36 | -0.02 | 0.86 | 0.36 | 0.86 | 0.36 | -0.14 | 0.27 | 0.11 | 0.11 | -0.19 | 0.31 | 0.27 |
| Bio13 |  |  |  |  |  |  |  |  |  |  |  |  |  | -0.42 | 0.74 | 0.76 | -0.43 | 0.76 | -0.43 | 0.44 | -0.19 | 0.07 | -0.03 | -0.42 | 0.22 | -0.08 |
| Bio14 |  |  |  |  |  |  |  |  |  |  |  |  |  |  | -0.85 | 0.16 | 0.99 | 0.16 | 0.99 | -0.82 | 0.47 | -0.10 | 0.11 | 0.18 | 0.13 | 0.50 |
| Bio15 |  |  |  |  |  |  |  |  |  |  |  |  |  |  |  | 0.32 | -0.86 | 0.32 | -0.86 | 0.64 | -0.48 | -0.05 | -0.18 | -0.43 | 0.01 | -0.31 |
| Bio16 |  |  |  |  |  |  |  |  |  |  |  |  |  |  |  |  | 0.15 | 1.00 | 0.15 | -0.18 | 0.05 | -0.11 | -0.09 | -0.45 | 0.28 | 0.30 |
| Bio17 |  |  |  |  |  |  |  |  |  |  |  |  |  |  |  |  |  | 0.15 | 1.00 | -0.83 | 0.47 | -0.10 | 0.11 | 0.19 | 0.13 | 0.51 |
| Bio18 |  |  |  |  |  |  |  |  |  |  |  |  |  |  |  |  |  |  | 0.15 | -0.18 | 0.05 | -0.11 | -0.09 | -0.45 | 0.28 | 0.30 |
| Bio19 |  |  |  |  |  |  |  |  |  |  |  |  |  |  |  |  |  |  |  | -0.83 | 0.47 | -0.10 | 0.11 | 0.19 | 0.13 | 0.51 |
| Altitude |  |  |  |  |  |  |  |  |  |  |  |  |  |  |  |  |  |  |  |  | -0.32 | 0.29 | 0.15 | 0.01 | -0.04 | -0.56 |
| Grazing |  |  |  |  |  |  |  |  |  |  |  |  |  |  |  |  |  |  |  |  |  | 0.00 | 0.06 | 0.02 | 0.10 | 0.13 |
| Residents |  |  |  |  |  |  |  |  |  |  |  |  |  |  |  |  |  |  |  |  |  |  | 0.06 | 0.34 | 0.02 | -0.17 |
| river |  |  |  |  |  |  |  |  |  |  |  |  |  |  |  |  |  |  |  |  |  |  |  | 0.33 | 0.02 | -0.03 |
| road |  |  |  |  |  |  |  |  |  |  |  |  |  |  |  |  |  |  |  |  |  |  |  |  | -0.10 | -0.07 |
| slope |  |  |  |  |  |  |  |  |  |  |  |  |  |  |  |  |  |  |  |  |  |  |  |  |  | 0.09 |

Supplementary table 4. The altitude, slope and aspect values of giant pandas, red pandas and livestock in Meigu Dafengding National Nature Reserve, China.

| Altitude (m) | | | Slope (°) | | | Aspect (°) | | |
| --- | --- | --- | --- | --- | --- | --- | --- | --- |
| Giant panda | Red panda | Livestock | Giant panda | Red panda | Livestock | Giant panda | Red panda | Livestock |
| 3197 | 2944 | 3408 | 22 | 18 | 23 | 128 | 70 | 150 |
| 3364 | 3240 | 3346 | 23 | 14 | 20 | 92 | 241 | 62 |
| 3392 | 2576 | 2942 | 50 | 7 | 8 | 140 | 62 | 166 |
| 3386 | 3486 | 3000 | 42 | 34 | 10 | 133 | 50 | 217 |
| 3382 | 3352 | 2978 | 36 | 32 | 8 | 117 | 10 | 157 |
| 3353 | 3386 | 2949 | 19 | 23 | 11 | 117 | 25 | 151 |
| 3313 | 2934 | 2833 | 19 | 7 | 15 | 60 | 337 | 33 |
| 3230 | 3185 | 3007 | 16 | 16 | 9 | 55 | 300 | 145 |
| 3204 | 3068 | 3397 | 26 | 24 | 8 | 359 | 200 | 79 |
| 3032 | 3549 | 3185 | 25 | 10 | 20 | 189 | 231 | 272 |
| 3087 | 3008 | 3323 | 7 | 31 | 17 | 117 | 103 | 245 |
| 3040 | 3239 | 2888 | 4 | 10 | 14 | 84 | 49 | 194 |
| 2966 | 3065 | 2952 | 13 | 33 | 14 | 139 | 57 | 36 |
| 3251 | 3039 | 3023 | 13 | 8 | 22 | 120 | 287 | 174 |
| 3161 | 2850 | 2405 | 1 | 31 | 10 | 239 | 49 | 120 |
| 3128 | 2728 | 3159 | 11 | 21 | 24 | 157 | 114 | 252 |
| 3051 | 2829 | 3027 | 20 | 21 | 25 | 229 | 0 | 250 |
| 3014 | 2995 | 3048 | 18 | 30 | 8 | 293 | 160 | 69 |
| 2998 | 3034 | 3521 | 10 | 27 | 11 | 217 | 48 | 64 |
| 2981 | 2741 | 3624 | 7 | 6 | 11 | 119 | 159 | 308 |
| 2949 | 2803 | 3604 | 11 | 17 | 18 | 151 | 81 | 318 |
| 3448 | 2756 | 3503 | 10 | 7 | 13 | 233 | 63 | 185 |
| 3424 | 2779 | 3461 | 33 | 12 | 10 | 219 | 22 | 317 |
| 3311 | 2719 | 3621 | 18 | 24 | 16 | 155 | 141 | 170 |
| 2839 | 2706 | 3640 | 27 | 19 | 15 | 72 | 117 | 108 |
| 2855 | 2567 | 3655 | 10 | 20 | 11 | 102 | 150 | 346 |
| 2924 | 2830 | 2910 | 9 | 25 | 8 | 129 | 163 | 288 |
| 2946 | 2618 | 2927 | 18 | 14 | 17 | 175 | 94 | 51 |
| 3012 | 2910 | 2890 | 10 | 28 | 8 | 139 | 184 | 119 |
| 3042 | 2496 | 3176 | 7 | 10 | 20 | 122 | 99 | 95 |
| 3132 | 2436 | 3129 | 25 | 17 | 7 | 144 | 187 | 193 |
| 2793 | 2649 | 3197 | 13 | 18 | 15 | 106 | 1 | 2 |
| 2808 | 2510 | 3050 | 17 | 9 | 15 | 76 | 304 | 297 |
| 2937 | 2635 | 3180 | 12 | 14 | 5 | 132 | 349 | 31 |
| 3061 | 2790 | 3031 | 4 | 10 | 22 | 183 | 356 | 215 |
| 3148 | 2691 | 2360 | 17 | 9 | 19 | 179 | 45 | 53 |
| 3273 | 2635 | 2442 | 31 | 25 | 4 | 95 | 351 | 68 |
| 2858 | 2701 | 2314 | 7 | 19 | 5 | 25 | 340 | 75 |
| 2879 | 2672 | 2544 | 14 | 25 | 17 | 36 | 314 | 163 |
| 2994 | 2677 | 2404 | 22 | 19 | 8 | 158 | 345 | 227 |
| 3043 | 2541 | 2248 | 15 | 43 | 7 | 100 | 204 | 220 |
| 3062 | 2514 | 2606 | 12 | 20 | 25 | 67 | 315 | 226 |
| 3180 | 2533 |  | 29 | 16 |  | 102 | 88 |  |
| 3227 | 2556 |  | 37 | 20 |  | 137 | 20 |  |
| 3336 |  |  | 23 |  |  | 24 |  |  |
| 3413 |  |  | 30 |  |  | 121 |  |  |
| 3371 |  |  | 15 |  |  | 350 |  |  |
| 3278 |  |  | 17 |  |  | 9 |  |  |
| 3517 |  |  | 16 |  |  | 320 |  |  |
| 3502 |  |  | 21 |  |  | 317 |  |  |
| 3485 |  |  | 22 |  |  | 24 |  |  |
| 3383 |  |  | 12 |  |  | 114 |  |  |
| 3296 |  |  | 27 |  |  | 357 |  |  |
| 3042 |  |  | 7 |  |  | 169 |  |  |
| 3111 |  |  | 30 |  |  | 232 |  |  |
| 3172 |  |  | 40 |  |  | 235 |  |  |
| 3296 |  |  | 25 |  |  | 231 |  |  |
| 3345 |  |  | 10 |  |  | 252 |  |  |
| 3452 |  |  | 19 |  |  | 245 |  |  |
| 3538 |  |  | 13 |  |  | 9 |  |  |
| 3428 |  |  | 20 |  |  | 191 |  |  |
| 3216 |  |  | 10 |  |  | 300 |  |  |
| 3105 |  |  | 9 |  |  | 213 |  |  |
| 2986 |  |  | 16 |  |  | 196 |  |  |
| 2889 |  |  | 14 |  |  | 194 |  |  |
| 2919 |  |  | 7 |  |  | 121 |  |  |
| 2919 |  |  | 21 |  |  | 155 |  |  |
| 2918 |  |  | 9 |  |  | 145 |  |  |
| 2952 |  |  | 16 |  |  | 42 |  |  |
| 2943 |  |  | 4 |  |  | 252 |  |  |
| 2924 |  |  | 13 |  |  | 123 |  |  |
| 2871 |  |  | 20 |  |  | 215 |  |  |
| 3039 |  |  | 23 |  |  | 176 |  |  |
| 3049 |  |  | 16 |  |  | 210 |  |  |
| 2965 |  |  | 23 |  |  | 183 |  |  |
| 3158 |  |  | 20 |  |  | 257 |  |  |
| 3148 |  |  | 20 |  |  | 257 |  |  |
| 3324 |  |  | 22 |  |  | 220 |  |  |
| 3236 |  |  | 33 |  |  | 306 |  |  |
| 3177 |  |  | 21 |  |  | 283 |  |  |
| 3164 |  |  | 14 |  |  | 216 |  |  |
| 3001 |  |  | 20 |  |  | 353 |  |  |
| 3156 |  |  | 19 |  |  | 328 |  |  |
| 3085 |  |  | 9 |  |  | 344 |  |  |
| 3102 |  |  | 17 |  |  | 334 |  |  |
| 2709 |  |  | 24 |  |  | 144 |  |  |
| 2690 |  |  | 15 |  |  | 185 |  |  |
| 2634 |  |  | 3 |  |  | 108 |  |  |
| 2514 |  |  | 27 |  |  | 105 |  |  |
| 2419 |  |  | 10 |  |  | 120 |  |  |
| 3078 |  |  | 20 |  |  | 203 |  |  |
| 3153 |  |  | 30 |  |  | 140 |  |  |
| 3251 |  |  | 6 |  |  | 184 |  |  |
| 3139 |  |  | 12 |  |  | 309 |  |  |
| 3129 |  |  | 12 |  |  | 257 |  |  |
| 3094 |  |  | 10 |  |  | 244 |  |  |
| 3092 |  |  | 15 |  |  | 284 |  |  |
| 3024 |  |  | 25 |  |  | 250 |  |  |
| 3352 |  |  | 25 |  |  | 1 |  |  |
| 3304 |  |  | 32 |  |  | 352 |  |  |
| 3215 |  |  | 15 |  |  | 27 |  |  |
| 3162 |  |  | 21 |  |  | 69 |  |  |
| 3181 |  |  | 24 |  |  | 124 |  |  |
| 3132 |  |  | 18 |  |  | 34 |  |  |
| 3078 |  |  | 14 |  |  | 40 |  |  |
| 3030 |  |  | 9 |  |  | 109 |  |  |
| 2990 |  |  | 10 |  |  | 94 |  |  |
| 2658 |  |  | 20 |  |  | 106 |  |  |
| 2848 |  |  | 13 |  |  | 195 |  |  |
| 2698 |  |  | 13 |  |  | 188 |  |  |
| 2885 |  |  | 25 |  |  | 239 |  |  |
| 2903 |  |  | 14 |  |  | 252 |  |  |
| 2831 |  |  | 12 |  |  | 179 |  |  |
| 2702 |  |  | 16 |  |  | 219 |  |  |
| 2611 |  |  | 16 |  |  | 121 |  |  |
| 3486 |  |  | 32 |  |  | 40 |  |  |
| 3361 |  |  | 10 |  |  | 53 |  |  |
| 3229 |  |  | 23 |  |  | 67 |  |  |
| 3171 |  |  | 32 |  |  | 63 |  |  |
| 2870 |  |  | 14 |  |  | 140 |  |  |
| 2755 |  |  | 23 |  |  | 113 |  |  |
| 3335 |  |  | 32 |  |  | 10 |  |  |
| 3386 |  |  | 17 |  |  | 39 |  |  |
| 3550 |  |  | 21 |  |  | 351 |  |  |
| 3586 |  |  | 11 |  |  | 335 |  |  |
| 3621 |  |  | 16 |  |  | 313 |  |  |
| 3604 |  |  | 16 |  |  | 150 |  |  |
| 3480 |  |  | 10 |  |  | 179 |  |  |
| 3255 |  |  | 12 |  |  | 130 |  |  |
| 3240 |  |  | 27 |  |  | 188 |  |  |
| 3227 |  |  | 20 |  |  | 261 |  |  |
| 3318 |  |  | 15 |  |  | 249 |  |  |
| 3515 |  |  | 6 |  |  | 241 |  |  |
| 3470 |  |  | 10 |  |  | 192 |  |  |
| 3428 |  |  | 26 |  |  | 249 |  |  |
| 3360 |  |  | 10 |  |  | 208 |  |  |
| 3433 |  |  | 6 |  |  | 189 |  |  |
| 3375 |  |  | 4 |  |  | 162 |  |  |
| 3516 |  |  | 11 |  |  | 167 |  |  |
| 3625 |  |  | 23 |  |  | 77 |  |  |
| 3586 |  |  | 15 |  |  | 108 |  |  |
| 3638 |  |  | 19 |  |  | 30 |  |  |
| 3655 |  |  | 14 |  |  | 127 |  |  |
| 3026 |  |  | 14 |  |  | 9 |  |  |
| 3024 |  |  | 14 |  |  | 318 |  |  |
| 2970 |  |  | 17 |  |  | 326 |  |  |
| 2936 |  |  | 8 |  |  | 288 |  |  |
| 2910 |  |  | 20 |  |  | 359 |  |  |
| 2934 |  |  | 9 |  |  | 339 |  |  |
| 2971 |  |  | 23 |  |  | 33 |  |  |
| 2976 |  |  | 13 |  |  | 25 |  |  |
| 2929 |  |  | 10 |  |  | 263 |  |  |
| 2917 |  |  | 14 |  |  | 207 |  |  |
| 2942 |  |  | 13 |  |  | 99 |  |  |
| 2909 |  |  | 3 |  |  | 22 |  |  |
| 2896 |  |  | 21 |  |  | 132 |  |  |
| 3276 |  |  | 31 |  |  | 141 |  |  |
| 3296 |  |  | 29 |  |  | 62 |  |  |
| 3347 |  |  | 26 |  |  | 35 |  |  |
| 3394 |  |  | 19 |  |  | 98 |  |  |
| 3395 |  |  | 25 |  |  | 77 |  |  |
| 3389 |  |  | 27 |  |  | 65 |  |  |
| 3360 |  |  | 24 |  |  | 116 |  |  |
| 3330 |  |  | 26 |  |  | 101 |  |  |
| 3037 |  |  | 16 |  |  | 112 |  |  |
| 3047 |  |  | 29 |  |  | 108 |  |  |
| 3091 |  |  | 26 |  |  | 118 |  |  |
| 3101 |  |  | 24 |  |  | 144 |  |  |
| 3156 |  |  | 26 |  |  | 138 |  |  |
| 3240 |  |  | 15 |  |  | 161 |  |  |
| 3291 |  |  | 31 |  |  | 145 |  |  |
| 3267 |  |  | 13 |  |  | 54 |  |  |
| 3204 |  |  | 24 |  |  | 215 |  |  |
| 3216 |  |  | 31 |  |  | 282 |  |  |
| 3191 |  |  | 35 |  |  | 313 |  |  |
| 3159 |  |  | 20 |  |  | 215 |  |  |
| 3104 |  |  | 16 |  |  | 219 |  |  |
| 2916 |  |  | 29 |  |  | 179 |  |  |
| 3043 |  |  | 32 |  |  | 140 |  |  |
| 3123 |  |  | 29 |  |  | 149 |  |  |
| 2871 |  |  | 15 |  |  | 78 |  |  |
| 2837 |  |  | 18 |  |  | 211 |  |  |
| 2893 |  |  | 10 |  |  | 281 |  |  |
| 2977 |  |  | 3 |  |  | 309 |  |  |
| 2894 |  |  | 15 |  |  | 345 |  |  |
| 2947 |  |  | 7 |  |  | 26 |  |  |
| 3002 |  |  | 22 |  |  | 302 |  |  |
| 3073 |  |  | 18 |  |  | 345 |  |  |
| 3096 |  |  | 8 |  |  | 315 |  |  |
| 3129 |  |  | 10 |  |  | 333 |  |  |
| 2973 |  |  | 17 |  |  | 41 |  |  |
| 2998 |  |  | 5 |  |  | 49 |  |  |
| 3075 |  |  | 7 |  |  | 86 |  |  |
| 3139 |  |  | 8 |  |  | 68 |  |  |
| 3216 |  |  | 16 |  |  | 100 |  |  |
| 3162 |  |  | 7 |  |  | 193 |  |  |
| 3119 |  |  | 5 |  |  | 322 |  |  |
| 3060 |  |  | 15 |  |  | 227 |  |  |
| 3065 |  |  | 19 |  |  | 3 |  |  |
| 3168 |  |  | 22 |  |  | 357 |  |  |
| 3214 |  |  | 5 |  |  | 292 |  |  |
| 3031 |  |  | 5 |  |  | 258 |  |  |
| 3031 |  |  | 21 |  |  | 9 |  |  |
| 3031 |  |  | 6 |  |  | 256 |  |  |
| 3034 |  |  | 5 |  |  | 242 |  |  |
| 2592 |  |  | 18 |  |  | 262 |  |  |
| 2658 |  |  | 3 |  |  | 278 |  |  |
| 2685 |  |  | 22 |  |  | 215 |  |  |
| 2764 |  |  | 22 |  |  | 215 |  |  |
| 2852 |  |  | 22 |  |  | 215 |  |  |
| 2905 |  |  | 22 |  |  | 215 |  |  |
| 3012 |  |  | 14 |  |  | 351 |  |  |
| 3122 |  |  | 19 |  |  | 295 |  |  |
| 3224 |  |  | 17 |  |  | 21 |  |  |
| 3205 |  |  | 5 |  |  | 135 |  |  |
| 3074 |  |  | 22 |  |  | 4 |  |  |
| 2970 |  |  | 9 |  |  | 55 |  |  |
| 3114 |  |  | 12 |  |  | 43 |  |  |
| 2994 |  |  | 15 |  |  | 171 |  |  |
| 2774 |  |  | 34 |  |  | 151 |  |  |
| 2830 |  |  | 14 |  |  | 209 |  |  |
| 2961 |  |  | 30 |  |  | 186 |  |  |
| 3141 |  |  | 6 |  |  | 122 |  |  |
| 3223 |  |  | 35 |  |  | 77 |  |  |
| 3312 |  |  | 13 |  |  | 189 |  |  |
| 3120 |  |  | 1 |  |  | 0 |  |  |
| 2952 |  |  | 14 |  |  | 45 |  |  |
| 3035 |  |  | 17 |  |  | 83 |  |  |
| 3136 |  |  | 14 |  |  | 40 |  |  |
| 3007 |  |  | 12 |  |  | 137 |  |  |
| 2854 |  |  | 13 |  |  | 18 |  |  |
| 2812 |  |  | 20 |  |  | 68 |  |  |
| 2763 |  |  | 24 |  |  | 52 |  |  |
| 2752 |  |  | 26 |  |  | 42 |  |  |
| 2669 |  |  | 24 |  |  | 43 |  |  |
| 2907 |  |  | 12 |  |  | 208 |  |  |
| 2962 |  |  | 19 |  |  | 68 |  |  |
| 3077 |  |  | 16 |  |  | 83 |  |  |
| 2924 |  |  | 8 |  |  | 209 |  |  |
| 3195 |  |  | 11 |  |  | 107 |  |  |
| 3120 |  |  | 27 |  |  | 46 |  |  |
| 3025 |  |  | 20 |  |  | 206 |  |  |
| 2980 |  |  | 25 |  |  | 159 |  |  |
| 2512 |  |  | 10 |  |  | 120 |  |  |
| 2874 |  |  | 12 |  |  | 93 |  |  |
| 2929 |  |  | 25 |  |  | 45 |  |  |
| 2800 |  |  | 19 |  |  | 229 |  |  |
| 2965 |  |  | 9 |  |  | 214 |  |  |
| 2617 |  |  | 16 |  |  | 335 |  |  |
| 2653 |  |  | 16 |  |  | 295 |  |  |
| 2838 |  |  | 20 |  |  | 329 |  |  |
| 2945 |  |  | 16 |  |  | 264 |  |  |
| 2930 |  |  | 27 |  |  | 336 |  |  |
| 2832 |  |  | 29 |  |  | 25 |  |  |
| 2524 |  |  | 8 |  |  | 182 |  |  |
| 2578 |  |  | 8 |  |  | 88 |  |  |
| 2609 |  |  | 16 |  |  | 147 |  |  |
| 2640 |  |  | 22 |  |  | 78 |  |  |
| 2810 |  |  | 24 |  |  | 50 |  |  |
| 2886 |  |  | 22 |  |  | 81 |  |  |
| 2846 |  |  | 34 |  |  | 189 |  |  |
| 2645 |  |  | 20 |  |  | 218 |  |  |
| 2877 |  |  | 16 |  |  | 332 |  |  |
| 2930 |  |  | 21 |  |  | 187 |  |  |
| 2936 |  |  | 17 |  |  | 216 |  |  |
| 2944 |  |  | 12 |  |  | 201 |  |  |
| 2948 |  |  | 10 |  |  | 307 |  |  |
| 2844 |  |  | 16 |  |  | 319 |  |  |
| 2751 |  |  | 18 |  |  | 39 |  |  |
| 2694 |  |  | 20 |  |  | 314 |  |  |
| 2595 |  |  | 24 |  |  | 296 |  |  |
| 2765 |  |  | 10 |  |  | 201 |  |  |
| 2854 |  |  | 7 |  |  | 171 |  |  |
| 3082 |  |  | 22 |  |  | 49 |  |  |
| 3059 |  |  | 14 |  |  | 252 |  |  |
| 3041 |  |  | 10 |  |  | 193 |  |  |
| 2607 |  |  | 20 |  |  | 109 |  |  |
| 2620 |  |  | 5 |  |  | 45 |  |  |
| 2654 |  |  | 9 |  |  | 162 |  |  |
| 2729 |  |  | 13 |  |  | 167 |  |  |
| 2671 |  |  | 26 |  |  | 265 |  |  |
| 2855 |  |  | 20 |  |  | 305 |  |  |
| 2957 |  |  | 8 |  |  | 270 |  |  |
| 3012 |  |  | 14 |  |  | 21 |  |  |
| 2970 |  |  | 18 |  |  | 274 |  |  |
| 2919 |  |  | 7 |  |  | 45 |  |  |
| 2909 |  |  | 5 |  |  | 293 |  |  |
| 2913 |  |  | 15 |  |  | 21 |  |  |
| 3069 |  |  | 28 |  |  | 31 |  |  |
| 3324 |  |  | 10 |  |  | 354 |  |  |
| 3324 |  |  | 9 |  |  | 311 |  |  |
| 3270 |  |  | 24 |  |  | 195 |  |  |
| 3138 |  |  | 11 |  |  | 99 |  |  |
| 3082 |  |  | 13 |  |  | 83 |  |  |
| 2838 |  |  | 17 |  |  | 125 |  |  |
| 2652 |  |  | 32 |  |  | 71 |  |  |
| 2625 |  |  | 11 |  |  | 278 |  |  |
| 2565 |  |  | 14 |  |  | 206 |  |  |
| 2518 |  |  | 17 |  |  | 108 |  |  |
| 2434 |  |  | 25 |  |  | 153 |  |  |
| 2468 |  |  | 23 |  |  | 159 |  |  |
| 2430 |  |  | 16 |  |  | 99 |  |  |
| 2289 |  |  | 13 |  |  | 49 |  |  |
| 2154 |  |  | 16 |  |  | 53 |  |  |
| 2244 |  |  | 10 |  |  | 73 |  |  |
| 2499 |  |  | 6 |  |  | 122 |  |  |
| 2476 |  |  | 20 |  |  | 115 |  |  |
| 2550 |  |  | 30 |  |  | 165 |  |  |
| 2623 |  |  | 23 |  |  | 129 |  |  |
| 2513 |  |  | 32 |  |  | 118 |  |  |
| 2441 |  |  | 8 |  |  | 45 |  |  |
| 2382 |  |  | 4 |  |  | 30 |  |  |
| 2342 |  |  | 16 |  |  | 121 |  |  |
| 2313 |  |  | 5 |  |  | 90 |  |  |
| 2727 |  |  | 12 |  |  | 165 |  |  |
| 2691 |  |  | 28 |  |  | 170 |  |  |
| 2623 |  |  | 19 |  |  | 150 |  |  |
| 2577 |  |  | 4 |  |  | 183 |  |  |
| 2582 |  |  | 4 |  |  | 153 |  |  |
| 2446 |  |  | 31 |  |  | 229 |  |  |
| 2380 |  |  | 33 |  |  | 182 |  |  |
| 2292 |  |  | 8 |  |  | 63 |  |  |
| 2260 |  |  | 22 |  |  | 145 |  |  |
| 2451 |  |  | 14 |  |  | 212 |  |  |
| 2432 |  |  | 21 |  |  | 136 |  |  |
| 2361 |  |  | 24 |  |  | 233 |  |  |
| 2316 |  |  | 27 |  |  | 216 |  |  |
| 2295 |  |  | 12 |  |  | 196 |  |  |
| 2329 |  |  | 6 |  |  | 180 |  |  |
| 2243 |  |  | 6 |  |  | 99 |  |  |
| 2232 |  |  | 0 |  |  | 45 |  |  |
| 2338 |  |  | 12 |  |  | 38 |  |  |
| 2525 |  |  | 16 |  |  | 332 |  |  |
| 2720 |  |  | 19 |  |  | 313 |  |  |
| 2724 |  |  | 16 |  |  | 301 |  |  |
| 2758 |  |  | 5 |  |  | 265 |  |  |
| 2704 |  |  | 14 |  |  | 165 |  |  |
| 2774 |  |  | 24 |  |  | 96 |  |  |
| 2719 |  |  | 20 |  |  | 79 |  |  |
| 2951 |  |  | 2 |  |  | 108 |  |  |
| 2931 |  |  | 12 |  |  | 124 |  |  |
| 2725 |  |  | 20 |  |  | 216 |  |  |
| 2701 |  |  | 10 |  |  | 35 |  |  |
| 2723 |  |  | 16 |  |  | 90 |  |  |
| 2511 |  |  | 7 |  |  | 11 |  |  |

Supplementary table 5. The contribution rates of each variable output by the Maxent model for giant pandas, red pandas and livestock under the different grazing condition in Meigu Dafengding National Nature Reserve, China.

| Giant pandas | | | | | Red pandas | | | |
| --- | --- | --- | --- | --- | --- | --- | --- | --- |
|  | Current grazing | Moderate grazing | Low grazing | No grazing | Current grazing | Moderate grazing | Low grazing | No  grazing |
| Grazing | 57.4% | 44.1% | 35.2% |  | 18.9% | 14.8% | 11.9% |  |
| Road | 13.8% | 14.9% | 14.8% | 22.7% | 10.7% | 5.8% | 13.6% | 8.9% |
| River | 5.7% | 9.9% | 10.5% | 12.8% | 5.1% | 3.7% | 8.4% | 9.8% |
| Vegetation | 1.8% | 5.4% | 6.5% | 10.2% | 16.8% | 20.5% | 19.2% | 24.1% |
| Altitude | 2.2% | 6.7% | 6.2% | 13.4% | 6.4% | 13.7% | 8.6% | 12.3% |
| aspect | 5.2% | 7.1% | 5.1% | 8.7% | 15.9% | 18.3% | 16.1% | 20.4% |
| slope | 5.9% | 7.7% | 8.5% | 8.5% | 5.4% | 5.8% | 4.9% | 8.9% |
| bio12 | 5.8% | 2.1% | 9.0% | 17.2% | 2.4% | 1.3% | 2.4% | 1.9% |
| bio2 | 2.2% | 2.1% | 4.2% | 6.5% | 18.4% | 16.1% | 14.9% | 13.7% |
